# Supplementary material for: Notch Signaling Facilitates In Vitro Generation of Cross-Presenting Classical Dendritic Cells
Source: Cell Rep. 2018 Jun 19;23(12):3658–3672.e6. doi: 10.1016/j.celrep.2018.05.068 (PMC6063084; doi:10.1016/j.celrep.2018.05.068)
Supplement: Document S1. Figures S1–S5 [file mmc1.pdf]

Cell Reports, Volume *23*

Supplemental Information

Notch Signaling Facilitates *In Vitro* Generation of Cross-Presenting Classical Dendritic Cells

Margaret E. Kirkling, Urszula Cytlak, Colleen M. Lau, Kanako L. Lewis, Anastasia Resteu, Alireza Khodadadi-Jamayran, Christian W. Siebel, Hélène Salmon, Miriam Merad, Aristotelis Tsirigos, Matthew Collin, Venetia Bigley, and Boris Reizis

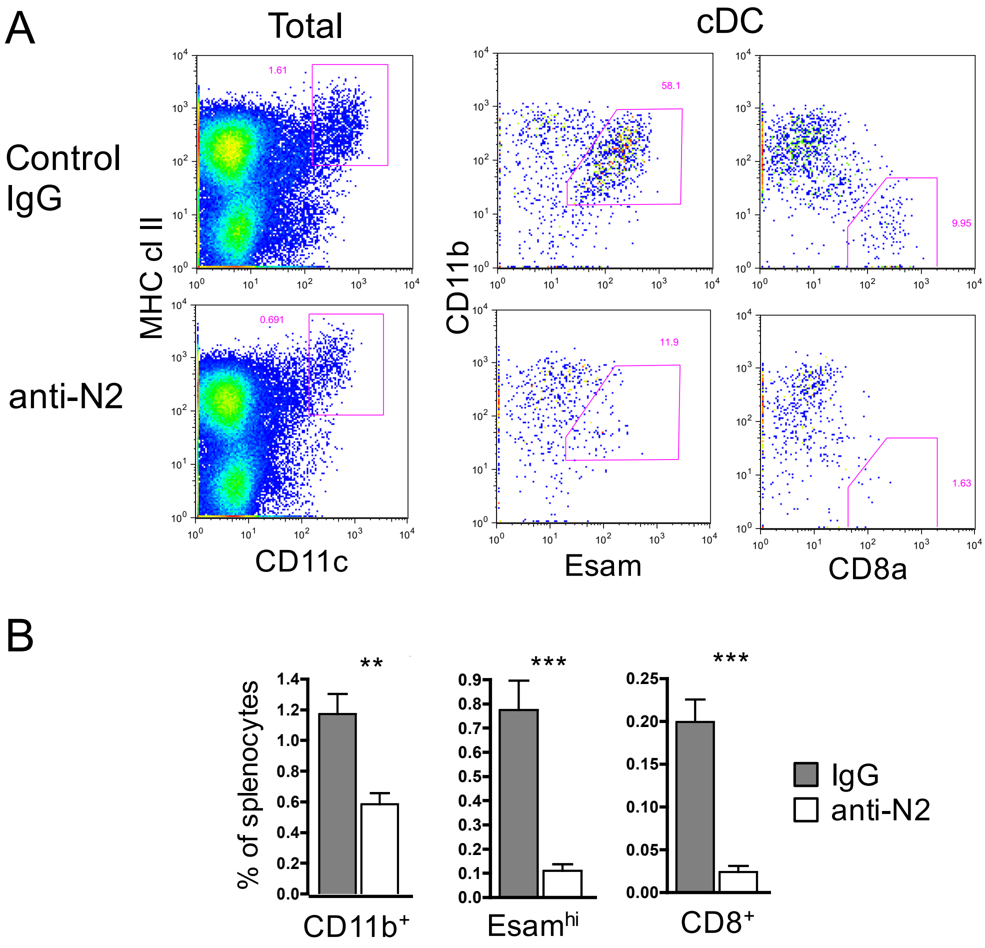

Figure S1. The effect of antibody-mediated Notch2 blockade in vivo. Related to Figure 1.
Wild-type animals were administered anti-N2 or control IgG 5 times every 4 days, and analyzed 4 days after the last administration.

A. Representative staining profiles of splenic DC. Shown are total splenocytes with $CD11c^{hi}$ MHC cl II^{+} cDC highlighted; and gated cDC with $CD11b^{+}$ $Esam^{hi}$ cDC2 and $CD11b^{-}$ $CD8\alpha^{+}$ cDC1 highlighted. Note the appearance of $CD11b^{-}$ $CD8\alpha^{-/lo}$ DC that were also previously observed upon genetic Notch2 deletion (Lewis et al., 2011; Satpathy et al., 2013) and likely represent cDC1 that lost CD8α expression.

B. The fraction of total $CD11b^{+}$ cDC2, $CD11b^{+}$ $Esam^{hi}$ cDC2 and $CD8\alpha^{+}$ cDC1 among total splenocytes. Data represent mean ± S.D. of 3 animals from one experiment; representative of 3 experiments.

Statistical significance was estimated using Student's *t*-test: ***, $p<0.001$; **, $p<0.01$.

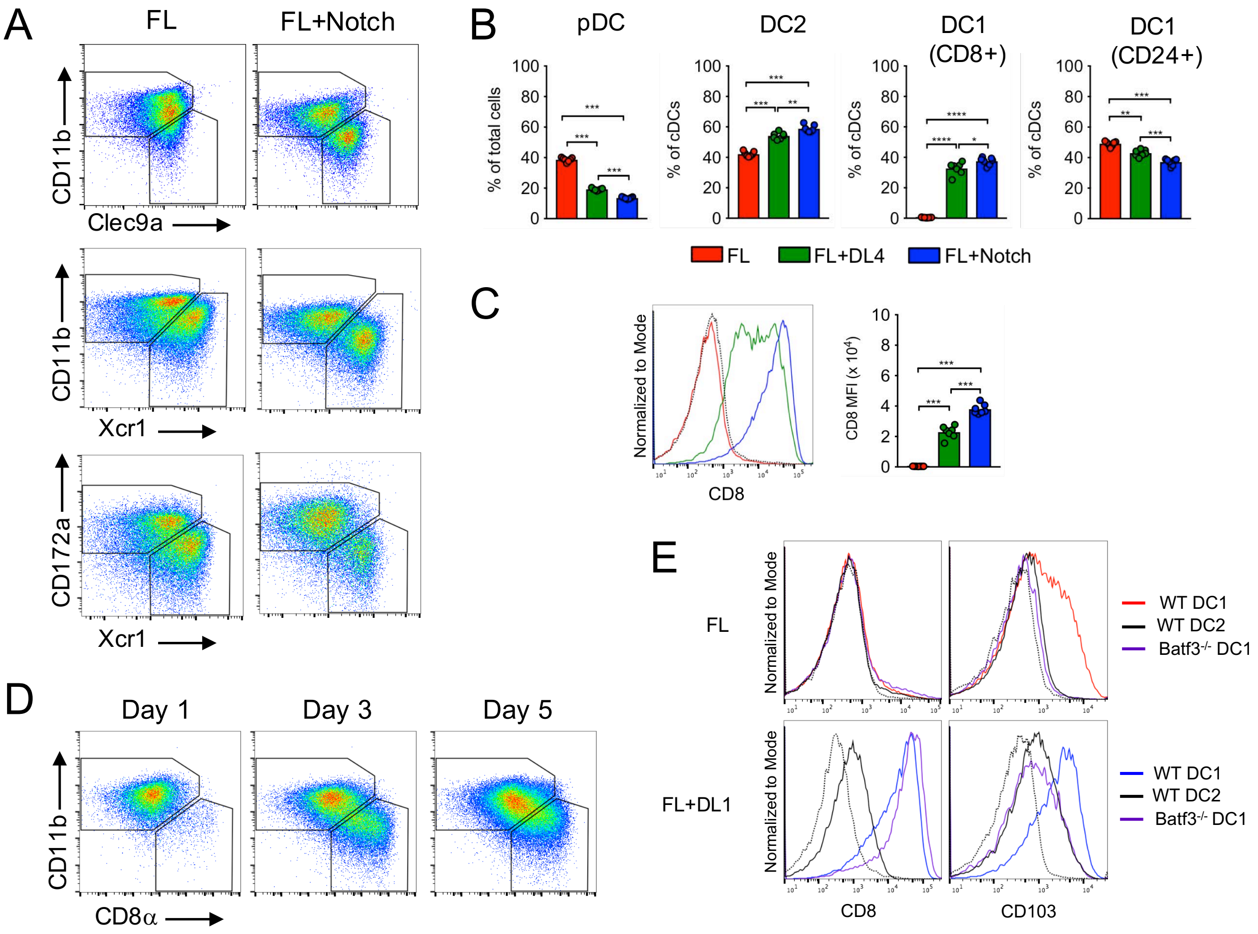

Figure S2. Additional characterization of bone marrow cell differentiation in FL-Notch cultures. Related to Figure 2.

Total murine BM cells were cultured in the presence of Flt3L alone (FL) or Flt3L with OP9 cells expressing the Notch ligand DL1 (FL+Notch). OP9 cells were added on day 3 unless indicated otherwise, and BM cells were analyzed on day 7 of differentiation.

A. Staining of cDC subsets with additional markers. Shown are representative plots of gated B220$^-$ CD11c$^+$ MHC II$^+$ cDC stained for cDC2 markers CD11b or CD172a and cDC1 markers Xcr1 or Clec9a.

B-C. The effect of Notch ligands on DC differentiation. BM cells were differentiated in FL cultures or co-cultures with OP9 expressing DL1 (FL+Notch) or DL4 (FL+DL4). Panel B shows the fraction of DC subsets on day 7. Panel C shows a representative expression profile and averaged mean fluorescence intensity (MFI) of CD8α on gated CD24$^+$ cDC1. Data points represent values in parallel BM cultures from individual mice; bars represent mean.

D. The timing of Notch signaling induction. OP9-DL1 cells were added at days 1, 3 or 5 of FL culture; shown are staining plots of gated B220$^-$ CD11c$^+$ MHC II$^+$ cDC with CD11b$^+$ CD8α$^-$ cDC2 and CD11b$^-$ CD8α$^+$ cDC1 highlighted.

E. The effect of Batf3 deletion on the surface phenotype of cDC1. Shown are representative expression profiles of CD8α and CD103 on gated CD24$^+$ cDC1 from control wild-type (WT) or Batf3-deficient mice. The expression on cDC2 is included as a control; dotted line represents negative staining control.

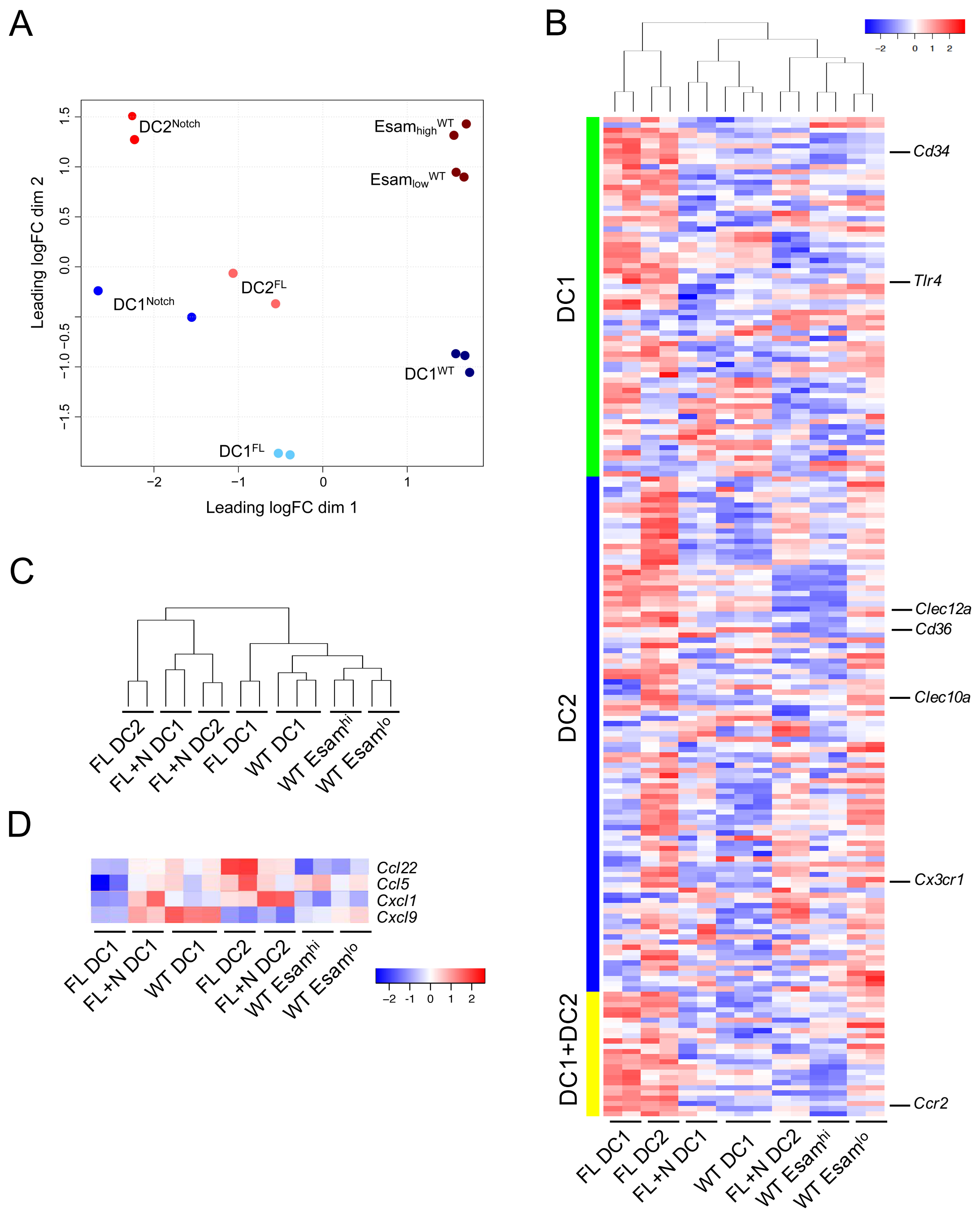

Figure S3. The expression profile of bone marrow-derived DC. Related to Figures 3 and 4.

Duplicate samples of sorted DC subsets from FL and FL-Notch cultures of primary bone marrow were analyzed by RNA-Seq.

A. Multidimensionality scaling (MDS) analysis of RNA-Seq profiles of culture-derived DC and primary splenic DC subsets from wild-type mice (WT). All samples are plotted on the 1st and 2nd dimension of MDS.

B. Heat map of Notch-repressed gene expression in cultured and primary DC subsets. Samples (labeled as in panel A) were hierarchically clustered by the expression of genes that were upregulated preferentially in Notch2-deficient cDC1, cDC2 or both cDC1+cDC2. Select genes are highlighted; color scale represents row Z-score.

C. Unsupervised clustering of cultured and primary splenic DC by the expression of chemokines (Table S4). Shown is the clustering dendrogram with individual replicates of the indicated samples.

D. Heat map of select chemokine expression in cultured and primary splenic DC as determined by RNA-Seq. Color scale represents row Z-score.

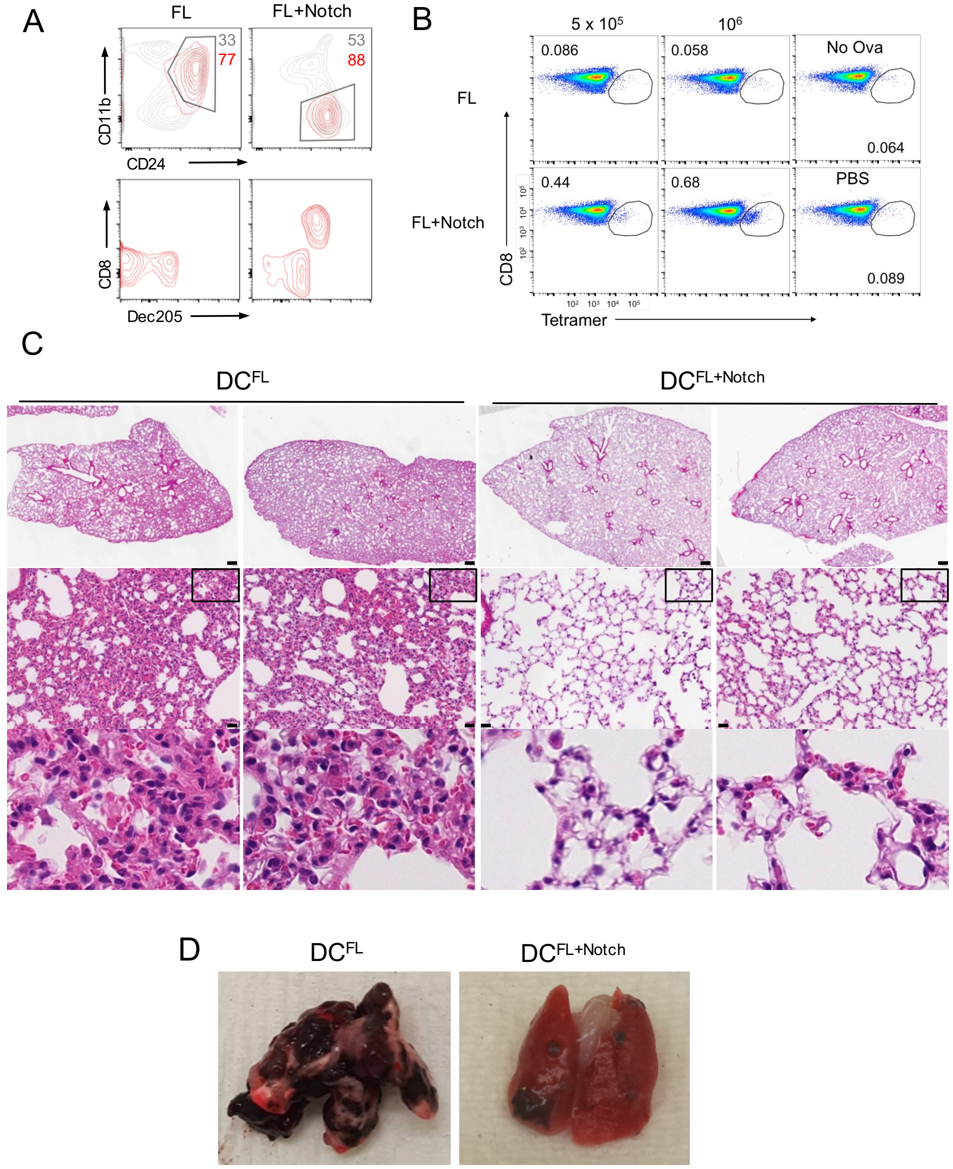

Figure S4. DC-mediated cross-priming and antitumor vaccination. Related to Figure 5.
DC from FL or FL+Notch cultures of primary murine BM were were incubated with OVA, and total DC or enriched cDC1 were injected i.v. into naive wild-type syngeneic recipient mice.
A. Representative cell populations used for in vivo transfer. Top panel shows the overlay of total DC (grey) and magnetically enriched cDC1 (red), with the corresponding $CD24^+$ cDC1 gate highlighted. Bottom panel shows the expression of CD8α and Dec205 in the enriched cDC1.
B. OVA-specific T cells in the spleens of mice that received the indicated number of OVA-pulsed total DC from FL or FL-Notch cultures. Shown are staining profiles of gated $CD44^+$ $TCRb^+$ activated T cells with the $CD8^+$ $tetramer^+$ cells highlighted. Control mice that received FL DC that were not pulsed with OVA (No OVA) or a mock injection (PBS) are also shown.
C. Lung histology of mice that received OVA-pulsed total DC and were subsequently challenged with OVA-expressing B16 melanoma cell line (B16-OVA). Lungs from two representative moribund DC^{FL} recipients (day 14 after B16-OVA injection) or healthy $DC^{FL\text{-}Notch}$ recipients (day 25 after B16-OVA injection) were fixed and stained with H&E. Bars: top row, 200 µm; middle row, 20 µm. Bottom row shows the areas highlighted in the middle row.
D. Gross morphology of the lungs from mice that received OVA-pulsed total DC and were subsequently challenged with B16-OVA. Unlike experiments in Fig. 5D and S4C, B16-OVA was delivered i.v. into the tail vein rather than into the retroorbital sinus. Shown are representative pictures of lungs excised from a moribund DC^{FL} recipient or a healthy $DC^{FL\text{-}Notch}$ recipient on day 28 after B16-OVA injection.

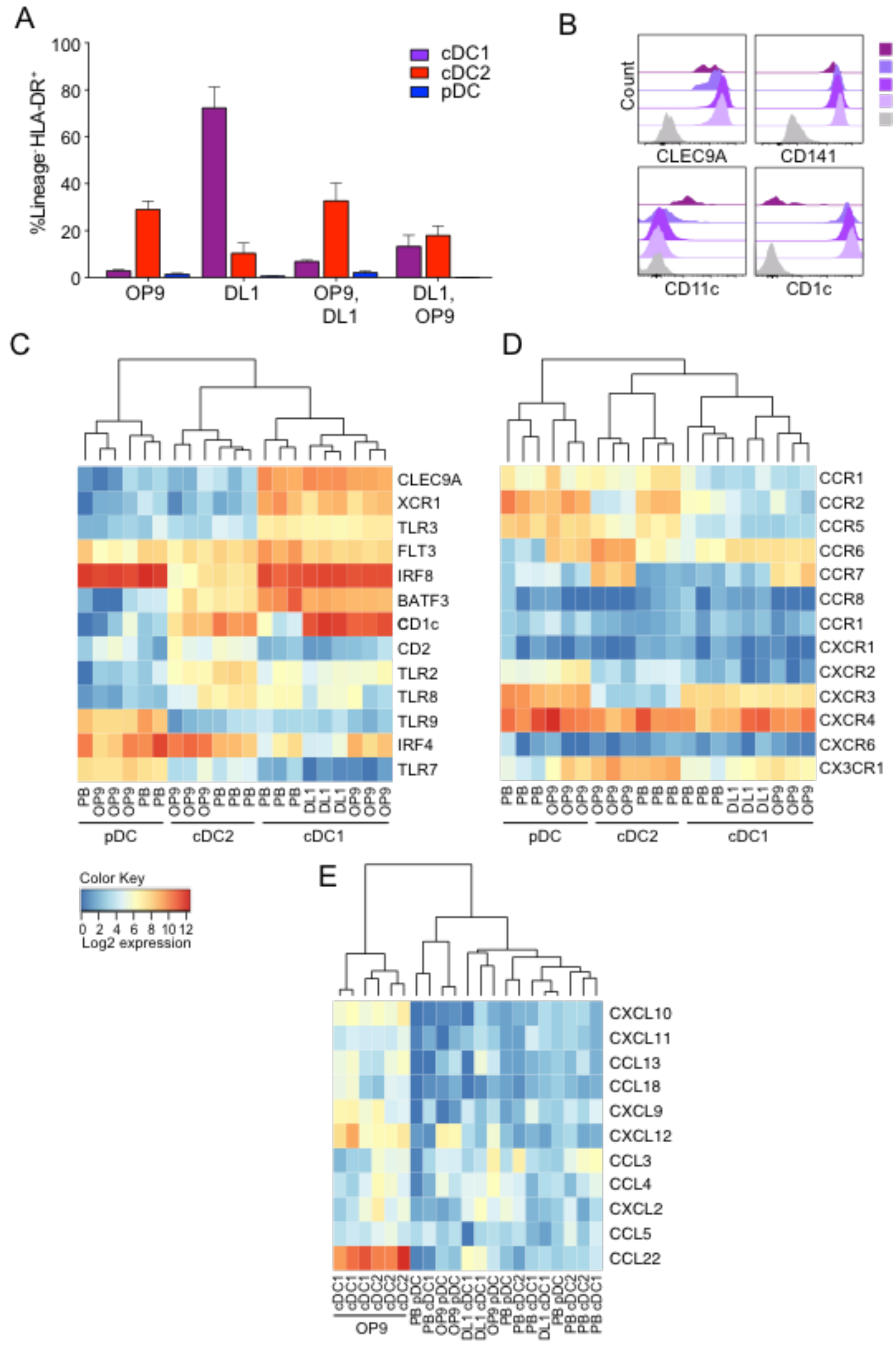

Figure S5, related to Figure 6. Development of cDC1 from human hematopoietic progenitors

Sorted CD34$^+$ stem/progenitor cells purified from the human BM were cultured for 2 weeks in the presence of a Flt3L-containing cytokine mix (FSGM) or on monolayers of control OP9, OP9-DL1 or OP9-DL4 cells.

A. The effect of the timing of OP9-DL1 addition. Cells were cultured on OP9 (n=9) or OP9-DL1 (DL1; n=7) for the entire culture period, or replated from OP9 to OP9-DL1 (OP9,DL1;n=3) or from OP9-DL1 to OP9 (DL1,OP9; n=2) after 1 week. Shown is the proportion of indicated DC subsets out of total Lin$^-$ HLA-DR$^+$ cells (mean ± S.E.M.)

B. The expression of DC markers. Shown are representative staining profiles of cDC1 from blood (PB) or culture with OP9, OP9-DL1 or OP9-DL4. Antigen expression is shown relative to isotype control (grey).

C-E. Heatmaps of DC subset specific surface antigen, TLR and transcription factor (**C**), chemokine receptor (**D**) or chemokine (**E**) gene expression in primary DC or DC cultured on OP9 or OP9-DL1 (DL1), as determined by NanoString nCounter analysis (Human Immunology_V2 panel plus 30 genes). Hierarchical clustering of samples by expression of indicated genes is shown. Color scales represent log2 expression.
