# Supplementary material for: Notch Signaling Facilitates In Vitro Generation of Cross-Presenting Classical Dendritic Cells
Source: Cell Rep. 2018 Jun 19;23(12):3658–3672.e6. doi: 10.1016/j.celrep.2018.05.068 (PMC6063084; doi:10.1016/j.celrep.2018.05.068)
Supplement: Document S2. Article plus Supplemental Information [file mmc7.pdf]

Resource

Cell Reports

Notch Signaling Facilitates *In Vitro* Generation of Cross-Presenting Classical Dendritic Cells

Graphical Abstract

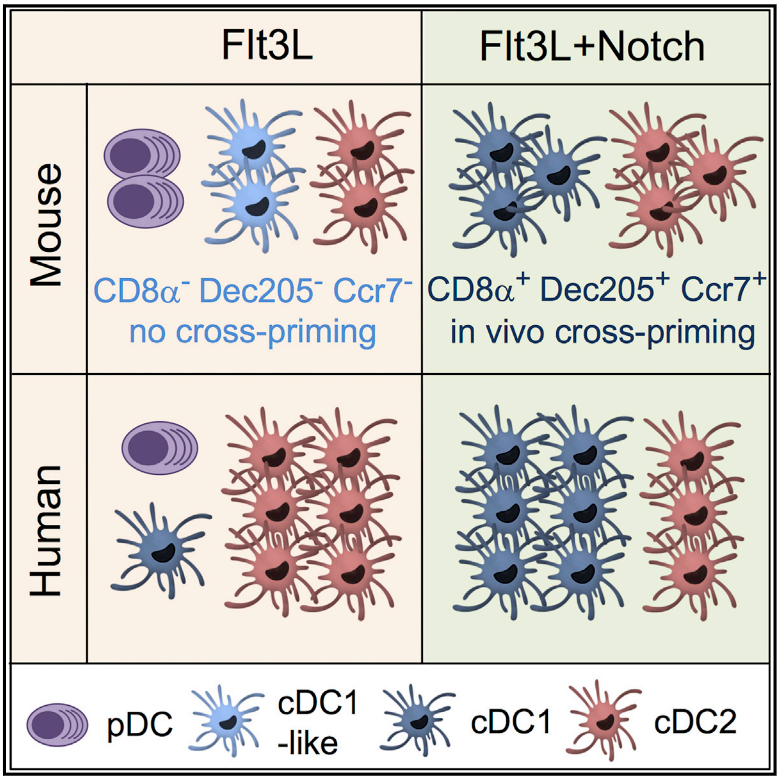

Authors

Margaret E. Kirkling, Urszula Cytlak, Colleen M. Lau, ..., Matthew Collin, Venetia Bigley, Boris Reizis

Correspondence

venetia.bigley@newcastle.ac.uk (V.B.), boris.reizis@nyumc.org (B.R.)

In Brief

Dendritic cells (DCs) are critical inducers of immune responses, but current methods to generate them *in vitro* are suboptimal. Kirkling et al. report that Notch signaling facilitates the generation of DCs that closely resemble their *in vivo* counterparts and show superior capacity to vaccinate against tumors *in vivo*.

Highlights

- DL1-Notch2 signaling induces differentiation of murine $CD8\alpha^+$ $CD103^+$ cDC1s *in vitro*
- Notch-induced cDC1s show improved expression profile and CCR7-dependent migration
- Notch-induced cDC1s mediate superior T cell cross-priming and antitumor vaccination
- DL1 signaling facilitates *in vitro* generation of human IRF8-dependent $CD141^+$ cDC1s

Kirkling et al., 2018, Cell Reports *23*, 3658–3672
June 19, 2018 © 2018 The Authors.
https://doi.org/10.1016/j.celrep.2018.05.068

CellPress

OPEN ACCESS
CellPress
Cell Reports
Resource

Notch Signaling Facilitates *In Vitro* Generation of Cross-Presenting Classical Dendritic Cells

Margaret E. Kirkling,[1,2,9] Urszula Cytlak,[3,9] Colleen M. Lau,[1] Kanako L. Lewis,[4] Anastasia Resteu,[3] Alireza Khodadadi-Jamayran,[1,5] Christian W. Siebel,[6] Hélène Salmon,[7] Miriam Merad,[7] Aristotelis Tsirigos,[1,5] Matthew Collin,[3,8] Venetia Bigley,[3,8,*] and Boris Reizis[1,4,10,*]
[1]Department of Pathology, New York University School of Medicine, New York, NY 10016, USA
[2]Graduate Program in Genetics and Development, Columbia University Medical Center, New York, NY 10032, USA
[3]Institute of Cellular Medicine, Newcastle University, Newcastle upon Tyne NE2 4HH, UK
[4]Department of Microbiology and Immunology, Columbia University Medical Center, New York, NY 10032, USA
[5]Applied Bioinformatics Laboratories, NYU School of Medicine, NY 10016, USA
[6]Genentech, Inc., 1 DNA Way, South San Francisco, CA 94080, USA
[7]Department of Oncological Science, Icahn School of Medicine at Mount Sinai, New York, NY 10028, USA
[8]Newcastle upon Tyne Hospitals NHS Foundation Trust, Freeman Road, Newcastle upon Tyne NE7 7DN, UK
[9]These authors contributed equally
[10]Lead Contact
*Correspondence: venetia.bigley@newcastle.ac.uk (V.B.), boris.reizis@nyumc.org (B.R.)
https://doi.org/10.1016/j.celrep.2018.05.068

SUMMARY

The IRF8-dependent subset of classical dendritic cells (cDCs), termed cDC1, is important for cross-priming cytotoxic T cell responses against pathogens and tumors. Culture of hematopoietic progenitors with DC growth factor FLT3 ligand (FLT3L) yields very few cDC1s (in humans) or only immature "cDC1-like" cells (in the mouse). We report that OP9 stromal cells expressing the Notch ligand Delta-like 1 (OP9-DL1) optimize FLT3L-driven development of cDC1s from murine immortalized progenitors and primary bone marrow cells. Co-culture with OP9-DL1 induced IRF8-dependent cDC1s with a phenotype ($CD103^+$ $Dec205^+$ $CD8\alpha^+$) and expression profile resembling primary splenic cDC1s. OP9-DL1-induced cDC1s showed preferential migration toward CCR7 ligands *in vitro* and superior T cell cross-priming and antitumor vaccination *in vivo*. Co-culture with OP9-DL1 also greatly increased the yield of IRF8-dependent $CD141^+$ cDC1s from human bone marrow progenitors cultured with FLT3L. Thus, Notch signaling optimizes cDC generation *in vitro* and yields authentic cDC1s for functional studies and translational applications.

INTRODUCTION

Dendritic cells (DCs) link innate and adaptive immunity by recognizing pathogens through pattern recognition receptors such as Toll-like receptors (TLRs) and recruiting diverse immune cells to orchestrate antigen (Ag)-specific adaptive responses (Pulendran, 2015; Steinman, 2012). Classical or conventional DCs (cDCs) are specialized Ag-presenting cells with a characteristic dendritic morphology, high major histocompatibility complex (MHC) class II expression, and a unique capacity for priming naive T cells. Upon Ag capture, cDCs upregulate chemotactic receptors such as CCR7, migrate from tissues into the T cell areas of regional lymphoid organs, secrete cytokines and chemokines, and present Ag to Ag-specific T cells. As such, cDCs hold great promise as cellular vaccines for eliciting Ag-specific immune responses, in particular to tumor antigens (Palucka and Banchereau, 2013).

In the mouse, cDCs are comprised of two main subsets: $CD8^+/CD103^+$ cDCs capable of Ag cross-presentation to $CD8^+$ T cells and $CD11b^+$ cDCs specialized in the presentation of exogenous Ag to $CD4^+$ T cells (Merad et al., 2013; Mildner and Jung, 2014; Schraml and Reis e Sousa, 2015). Both subsets are conserved in humans (Haniffa et al., 2015) and have recently been designated as cDC1 and cDC2, respectively (Guilliams et al., 2014). All DCs, including cDCs and the related lineage of interferon-producing plasmacytoid DCs (pDCs), develop in the bone marrow (BM) in a process driven mainly by the cytokine FLT3 ligand (FLT3L). Progenitors committed to cDC subsets (pre-DCs) exit the BM and undergo terminal differentiation in peripheral lymphoid organs and tissues. The development of DC subsets is driven by several transcription factors, such as IRF8, which is absolutely required for cDC1 differentiation in mice (Aliberti et al., 2003; Sichien et al., 2016) and in humans (Bigley et al., 2017; Hambleton et al., 2011). Additional factors, such as BATF3 and other BATF family members, cooperate with IRF8 to facilitate optimal development of cDC1s (Hildner et al., 2008; Murphy et al., 2016). In addition to these cell-intrinsic factors, terminal cDC differentiation in the periphery is guided by tissue-specific signals, such as lymphotoxin-β and Notch.

Notch is an evolutionarily conserved pathway of cell-cell communication that informs cells of their surroundings and, thereby, guides their differentiation. Vertebrate Notch receptors (NOTCH1–4) transmit signals from membrane-bound ligands of the Delta-like (DL) and Jagged (Jag) families through the

 © 2018 The Authors.
This is an open access article under the CC BY license (http://creativecommons.org/licenses/by/4.0/).

common transcription factor CSL (also called RBPJ). Notch signaling plays an essential role in the development of immune cell types that differentiate in distinct anatomical niches. For instance, DL4-NOTCH1 and DL1-NOTCH2 signaling is required for the specification of T cells in the thymus and of marginal zone (MZ) B cells in the spleen, respectively (Radtke et al., 2013). Indeed, co-culture of stem/progenitor cells with a murine stromal cell line OP9 expressing DL1 (OP9-DL1) has become a standard approach to generate T cells in vitro (Schmitt et al., 2004; Mohtashami et al., 2016). Using DC-specific gene targeting, we have established the role of NOTCH2 receptor signaling in the differentiation of a cDC2 subset in the spleen and intestine (Caton et al., 2007; Lewis et al., 2011). In particular, splenic cDC2 contains a lymphotoxin-β- and NOTCH2-RBPJ-dependent Esam^{hi} subset that is required for optimal CD4^{+} T cell priming. These studies also revealed the reduction of Notch2-deficient splenic CD8α^{+} cDC1s (Lewis et al., 2011), which was subsequently ascribed to their impaired differentiation and aberrant phenotype (Satpathy et al., 2013). Finally, DL1 expressed on fibroblasts has been identified as the relevant ligand of NOTCH2 on splenic cDCs (Fasnacht et al., 2014). Thus, NOTCH2 signaling mediated by DL ligands on stromal cells controls the phenotypic and functional differentiation of both cDC subsets.

Because primary DCs (particularly cDC1s) are rare *in vivo*, their study and translational applications require methods to generate functional DC subsets *in vitro*. Commonly used cultures of primary BM with the cytokine granulocyte-monocyte colony-stimulating factor (GM-CSF) produce a mixture of cDC2-like cells and macrophages (Helft et al., 2015) but no cDC1s. Cultures of murine BM supplemented with the physiological cytokine FLT3L produce a mixture of pDCs, cDC2s and cDC1-like cells (Naik et al., 2005). The latter express appropriate transcription factors, including IRF8, but have an abnormal phenotype, including a lack of key cDC1 markers (e.g., CD8α, CD103, and Dec205) and aberrant expression of cDC2 markers (e.g., CD11b). Human hematopoietic stem and progenitor cells cultured with FLT3L and other cytokines and/or stromal cells can produce CD141^{+} cDC1s with the expected expression profile and *in vitro* functional properties (Balan et al., 2014; Lee et al., 2015; Poulin et al., 2010; Proietto et al., 2012). However, the yield of cDC1s has been very low in all reported protocols. Thus, new approaches are necessary to produce the full spectrum and high numbers of fully differentiated DCs, particularly of functional cDC1s.

Given the important role of Notch signaling in cDC differentiation *in vivo*, we hypothesized that it would facilitate cDC differentiation *in vitro*. We now report that combination of FLT3L-driven differentiation of murine hematopoietic progenitors with the well-established OP9-DL1 system produces optimally differentiated cDC subsets, including *bona fide* CD8α^{+} Dec205^{+} cDC1. The resulting cDC1s showed improved migration properties and superior T cell cross-priming capacity *in vivo*. Furthermore, co-culture of human hematopoietic progenitors with OP9-DL1 enhanced the generation of functional human cDC1s. These results emphasize the key role of Notch signaling in terminal cDC differentiation and facilitate the generation of functional cDC1s for translational applications.

RESULTS

Notch Signaling Enables cDC1 Differentiation of Immortalized DC Progenitors

To optimize DC production *in vitro*, we initially studied the differentiation of DC progenitors that were conditionally immortalized with the estrogen-dependent HoxB8 oncogene (Redecke et al., 2013). In this system, progenitors can be grown indefinitely in the presence of FLT3L and estrogen, whereas estrogen withdrawal induces spontaneous FLT3L-driven DC differentiation within ~7 days. Although HoxB8-FL (FLT3L) cells were originally reported to produce all DC subsets, we never observed the production of CD24^{+} or CD8α^{+} cDC1s from the original HoxB8-FL line or from any newly derived lines (Figure 1A). To mimic Notch signals received by committed cDC progenitors *in vivo*, we differentiated HoxB8-FL cells for 3 days to initiate DC development and then plated them on a monolayer of OP9-DL1 cells or control GFP-transduced OP9 cells for the last 4 days of culture. Control OP9 cells inhibited the development of B220^{+} pDCs and yielded only CD11b^{hi} CD24^{-} CD8α^{-} cDC2s in lower numbers (Figure 1A and data not shown). Co-culture with OP9-DL1 largely abolished pDC development and reduced the yield of cDC2s; however, it induced the generation of a distinct cDC subset with the CD11b^{-} CD24^{+} CD8α^{+} phenotype of cDC1s (Figures 1A and 1B).

To confirm that the observed DL1-driven cDC1 generation is NOTCH2-dependent, we used an antibody (Ab) that blocks the activation of NOTCH2 (anti-N2) (Wu et al., 2010). The administration of this anti-N2 Ab *in vivo* recapitulated the effects of DC-specific NOTCH2 blockade, including ablation of splenic Esam^{hi} cDC2s and of intestinal CD11b^{+} CD103^{+} cDCs and loss of CD8α^{+} splenic cDC1s (Figure S1 and data not shown). The addition of anti-N2 to HoxB8-FL co-cultures with OP9-DL1 did not rescue pDC loss (Figure 1C), further suggesting that this loss is caused by OP9 cells independent of DL1 expression. On the other hand, anti-N2 reduced the development of cDC2s and largely abolished the development of cDC1s (Figure 1C). To test the function of NOTCH2 ligands, we used OP9 cells transduced with DL4 (OP9-DL4), which are as efficient as OP9-DL1 in driving T cell development (Mohtashami et al., 2016). We found that OP9-DL4 were able to induce cDC1 differentiation of HoxB8-FL cells, but less efficiently than OP9-DL1 (Figure 1D). Collectively, these data show that DL1-NOTCH2 signaling can elicit *de novo* generation of cDC1s from immortalized DC progenitors.

Notch Signaling Induces Optimal cDC1 Differentiation of BM Progenitors

Having established the OP9-DL1 co-culture system of DC differentiation, we applied it to the cultures of primary BM. Total BM cells were either cultured for 7 days using a standard DC differentiation protocol in FLT3L-containing medium or transferred on day 3 to monolayers of OP9-DL1 in the continued presence of FLT3L (hereafter referred to as FL or FL-Notch cultures, respectively). As described previously (Naik et al., 2005), DCs generated in FL cultures (DC^{FL}) comprised B220^{+} pDCs, CD11b^{+} CD24^{-} cDC2s, and CD11b$^{+/low}$ CD24^{+} cDC1-like cells (Figure 2A). Co-culture with control OP9 cells impaired the

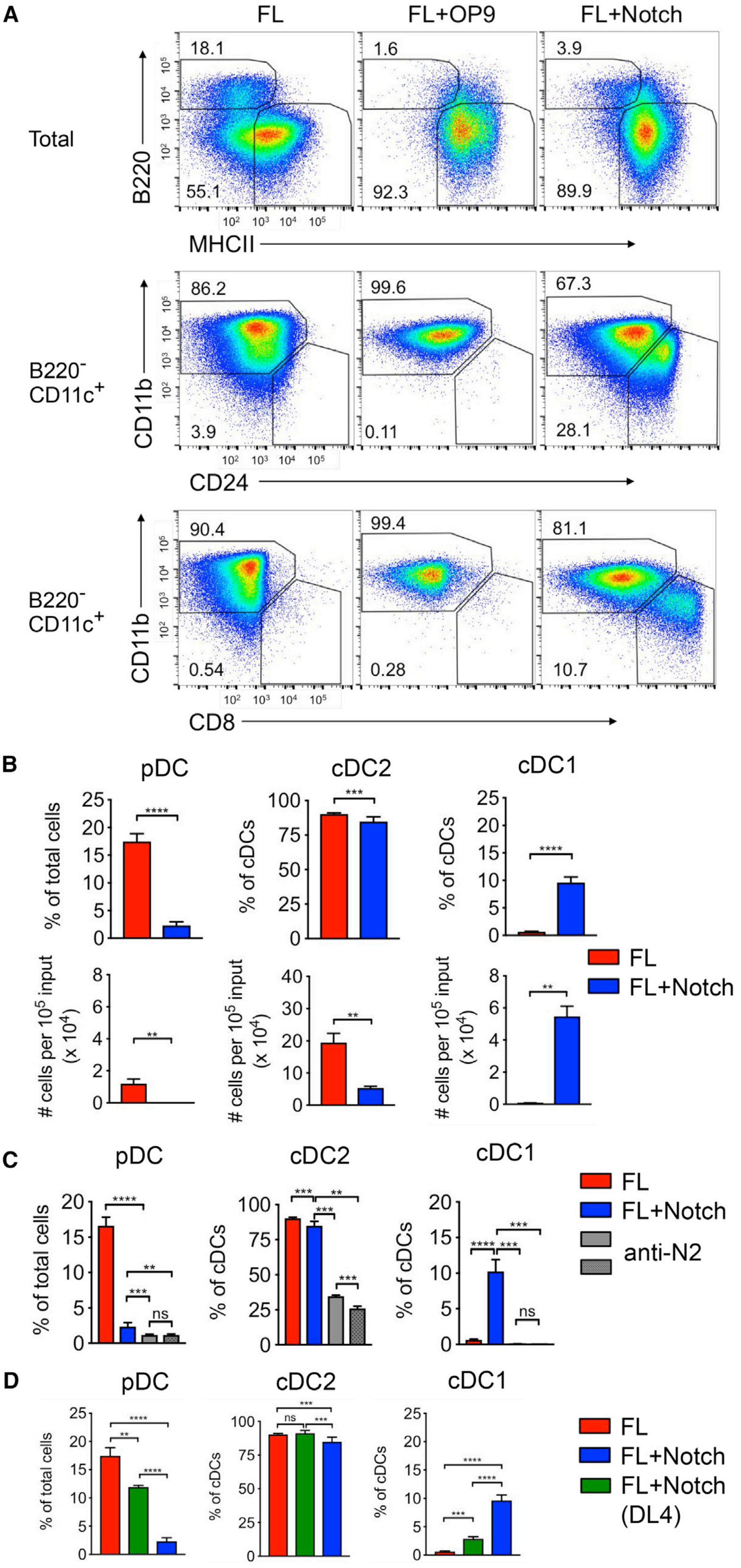

Figure 1. DL1-NOTCH2 Signaling Induces Differentiation of cDC1s from a DC Progenitor Cell Line

The HoxB8-FL cell line was induced to differentiate *in vitro* by estrogen withdrawal in the presence of FLT3L alone (FL), FLT3L with control OP9 cells (FL+OP9), or FLT3L with OP9 cells expressing the Notch ligand DL1 (FL+Notch). OP9 cells were added on day 3, and HoxB8-FL cells were analyzed on day 7 of differentiation.

(A) Representative staining plots of differentiated HoxB8-FL cells. The top row shows total live cells with $B220^{+}$ MHC class II^{lo} pDCs and $B220^{-}$ MHC class II^{hi} cDCs highlighted; the other rows show gated cDCs with $CD11b^{hi}$ cDC2s and $CD11b^{lo/-}$ cDC1s highlighted.

(B) The subset composition of differentiated HoxB8-FL cells. Shown are fractions of pDCs (of total live cells) and cDC subsets (of gated cDCs) and the absolute number of these subsets per 10^5 initial undifferentiated cells. Data represent mean ± SD of 6 parallel cultures, representative of 3 experiments.

(C) The effect of NOTCH2 blockade on HoxB8-FL cell differentiation. HoxB8-FL cells were differentiated in FL+Notch cultures in the presence of anti-NOTCH2 blocking Ab (anti-N2) at 50 ng/ml (gray open bars) or 500 ng/ml (gray textured bars); the fractions of DC subsets are shown as above. Data represent mean ± SD of 5 parallel cultures.

(D) The effect of Notch ligands on HoxB8-FL cell differentiation. HoxB8-FL cells were differentiated in co-cultures with OP9 cells expressing the Notch ligand DL1 or DL4; the fractions of DC subsets are shown as above. Data represent mean ± SD of 6 parallel cultures.

Statistical significance: ****$p < 0.0001$; ***$p < 0.001$; **$p < 0.01$; ns, not significant.

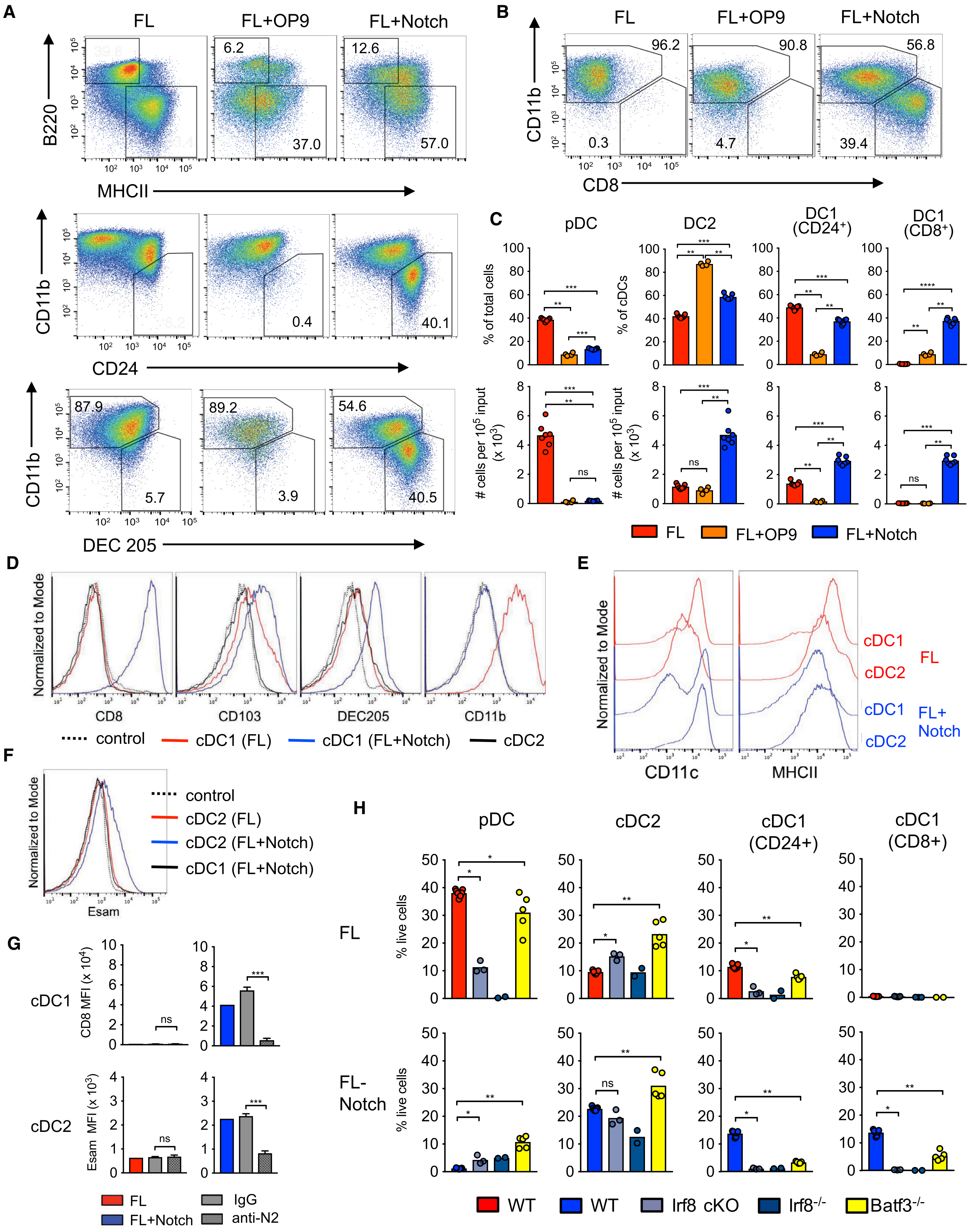

(legend on next page)

generation of pDCs and cDC1-like cells, yielding primarily cDC2s (Figures 2A–2C). In contrast, co-culture with OP9-DL1 inhibited pDC but not cDC development; moreover, the resulting DC population ($DC^{FL\text{-}Notch}$) contained two fully resolved subsets, including a distinct $CD11b^{-}$ $CD24^{+}$ cDC1 population that expressed Dec205 and CD8α (Figures 2A–2C). In contrast to cDC1-like cells from FL cultures ($cDC1^{FL}$), $CD24^{+}$ cDC1s from FL-Notch cultures ($cDC1^{FL\text{-}Notch}$) downregulated CD11b, acquired expression of Dec205 and CD8α, and upregulated CD103 (Figure 2D). Staining for additional subset markers revealed a similarly improved resolution of the two cDC subsets in FL-Notch cultures (Figure S2A). In particular, $cDC2^{FL}$ expressed low levels of the cDC1 markers Xcr1 and Clec9a, which were reduced in $cDC2^{FL\text{-}Notch}$; conversely, $cDC1^{FL\text{-}Notch}$ expressed Xcr1 and Clec9a but lacked the cDC2 markers CD11b and CD172a/Sirpα (Figure S2A). All $DC^{FL\text{-}Notch}$ had higher levels of CD11c and reduced levels of surface MHC class II; the latter was particularly evident in cDC1s (Figure 2E). This is consistent with the phenotype of resident cDCs in lymphoid organs, which have higher CD11c and lower MHC class II surface levels than migratory tissue-derived cDCs. Furthermore, cDC2s from FL-Notch cultures showed induction of Esam, a marker of NOTCH2-dependent splenic cDC2s (Figure 2F). Thus, DL1-NOTCH2 signaling refines the phenotypes of BM-derived cDC subsets and brings them closer to those of the primary resident cDC in lymphoid organs.

The induction of CD8α on cDC1s and of Esam on cDC2s was blocked by anti-N2 Ab (Figure 2G), confirming NOTCH2 as the relevant receptor. Both DL1- and DL4-expressing OP9 cells were able to induce differentiation of $CD8\alpha^{+}$ cDC1s; however, DL1 induced higher levels of CD8α expression than DL4 (Figures S2B and S2C). Addition of OP9-DL1 at an earlier (day 1) or later (day 5) time point was less efficient (Figure S2D), suggesting that a durable Notch signal delivered after the initial DC lineage commitment provides optimal DC subset resolution.

As expected, global or DC-specific deletion of *Irf8* spared cDC2s, impaired pDCs, and fully abolished the development of cDC1-like cells in FL cultures (Figure 2H). On the other hand, deletion of *Batf3* only mildly affected $cDC1^{FL}$ development. cDC1s in FL-Notch cultures showed the same strict dependence on *Irf8* but also a stronger dependence on *Batf3* (Figure 2H). Furthermore, *Batf3* deletion reduced the expression of CD103 on cDC1s, as described previously (Jackson et al., 2011); this effect was particularly prominent in $CD103^{hi}$ $cDC1^{FL\text{-}Notch}$ (Figure S2E). Altogether, these results suggest that FL-Notch cultures of primary BM yield cDC1 cells with the appropriate phenotype and genetic requirements.

Notch Signaling Optimizes the Global Expression Profile of *In Vitro*-Derived DCs

To further explore the effect of DL1-Notch signaling on *in vitro*-derived DCs, we sorted cDC1s and cDC2s from FL and FL-Notch cultures and interrogated their expression profiles by global mRNA sequencing (RNA-seq). The resulting expression profiles were merged with those of primary splenic cDCs (Lau et al., 2016; Table S1) and compared using multidimensionality scaling (MDS) analysis. As expected, the first dimension separated culture-derived DCs from primary DCs (Figure S3A), whereas the second and third dimensions separated DC subsets (Figure 3A). By both the second and third dimension, $cDC2^{FL\text{-}Notch}$ clustered closer than $cDC2^{FL}$ to primary cDC2 samples, particularly to the NOTCH2-dependent $Esam^{hi}$ subset (Figure 3A). Moreover, $cDC1^{FL\text{-}Notch}$ clustered much closer to primary cDC1 compared with cDC1-like cells from FL cultures (Figure 3A).

Pairwise comparison between cDC1 and cDC2 for each culture condition showed a greater divergence of the two subsets in FL-Notch cultures (Figure 3B). In particular, characteristic markers of cDC1 (*Xcr1*, *Clec9a*, *CD36*, and *Tlr11*) and cDC2 (*Itgam* and *Lyz2*) showed greater differential expression. Pairwise comparison between FL and FL-Notch cultures for each subset (Figure 3C; Table S2) showed that $cDC2^{FL\text{-}Notch}$ induced the expression of NOTCH2-dependent genes overexpressed in $Esam^{hi}$ cDC2 (Lewis et al., 2011; Satpathy et al.,

Figure 2. DL1-NOTCH2 Optimizes the Differentiation of cDC1s from the BM

Total murine BM cells were cultured in the presence of FLT3L alone (FL), FLT3L with control OP9 cells (FL+OP9) or FLT3L with OP9 cells expressing Notch ligand DL1 (FL+Notch). OP9 cells were added on day 3, and BM cells were analyzed on day 7 of differentiation.

(A) Representative staining plots of differentiated BM cells. The top row shows total live cells with $B220^{+}$ $CD11c^{lo}$ pDCs and $B220^{-}$ $CD11c^{hi}$ cDCs highlighted; the other rows show gated cDCs with $CD11b^{hi}$ cDC2s and/or $CD11b^{-}$ cDC1s highlighted.

(B) The expression of CD8α on gated cDCs in BM cultures differentiated as in (A).

(C) The subset composition of differentiated BM cells. Shown are fractions of pDCs (of total live cells) and cDC subsets (of gated $B220^{-}$ $CD11c^{+}$ MHC class II^{+} cDCs) and the absolute number of these subsets per 10^{5} initial BM cells. cDC1s were defined either as $CD24^{+}$ or $CD8\alpha^{+}$. Data points represent values from BM cultures of individual mice pooled from 2 experiments; bars represent mean.

(D) Representative expression of the indicated surface markers on gated $CD24^{+}$ cDC1s from FL or FL+Notch cultures. The expression of cDC1 markers on cDC2s is included as a control.

(E) Representative expression of CD11c and MHC class II on DC subsets from FL or FL+Notch cultures. Subsets were gated, omitting the marker that is shown in each case.

(F) Representative expression of Esam on cDC2s from FL or FL+Notch cultures. The expression on cDC1s is included as a control; the dotted line represents negative staining.

(G) The effect of NOTCH2 blockade on the expression of CD8α on cDC1s and of Esam on cDC2s. BM cells were differentiated in FL or FL+Notch cultures in the presence of control immunoglobulin G (IgG) or anti-N2; the fluorescence intensity of marker expression in the indicated subsets is shown. Data represent mean ± SD of 5 parallel cultures for anti-N2 and IgG and 9 cultures pooled from 2 experiments for controls.

(H) The effect of transcription factor deletion on DC differentiation. BM from control wild-type (WT) mice or mice with DC-specific deletion of *Irf8* (*Irf8* conditional knockout, cKO) or with germline deletion of *Irf8* or *Batf3* were cultured in FL or FL-Notch cultures. Shown is the fraction of the indicated DC subsets among total live cells; data points represent values from individual mice pooled from three (for *Irf8*) or two (for *Batf3*) experiments; bars represent mean.

Statistical significance: ***$p < 0.001$, **$p < 0.01$, *$p < 0.05$.

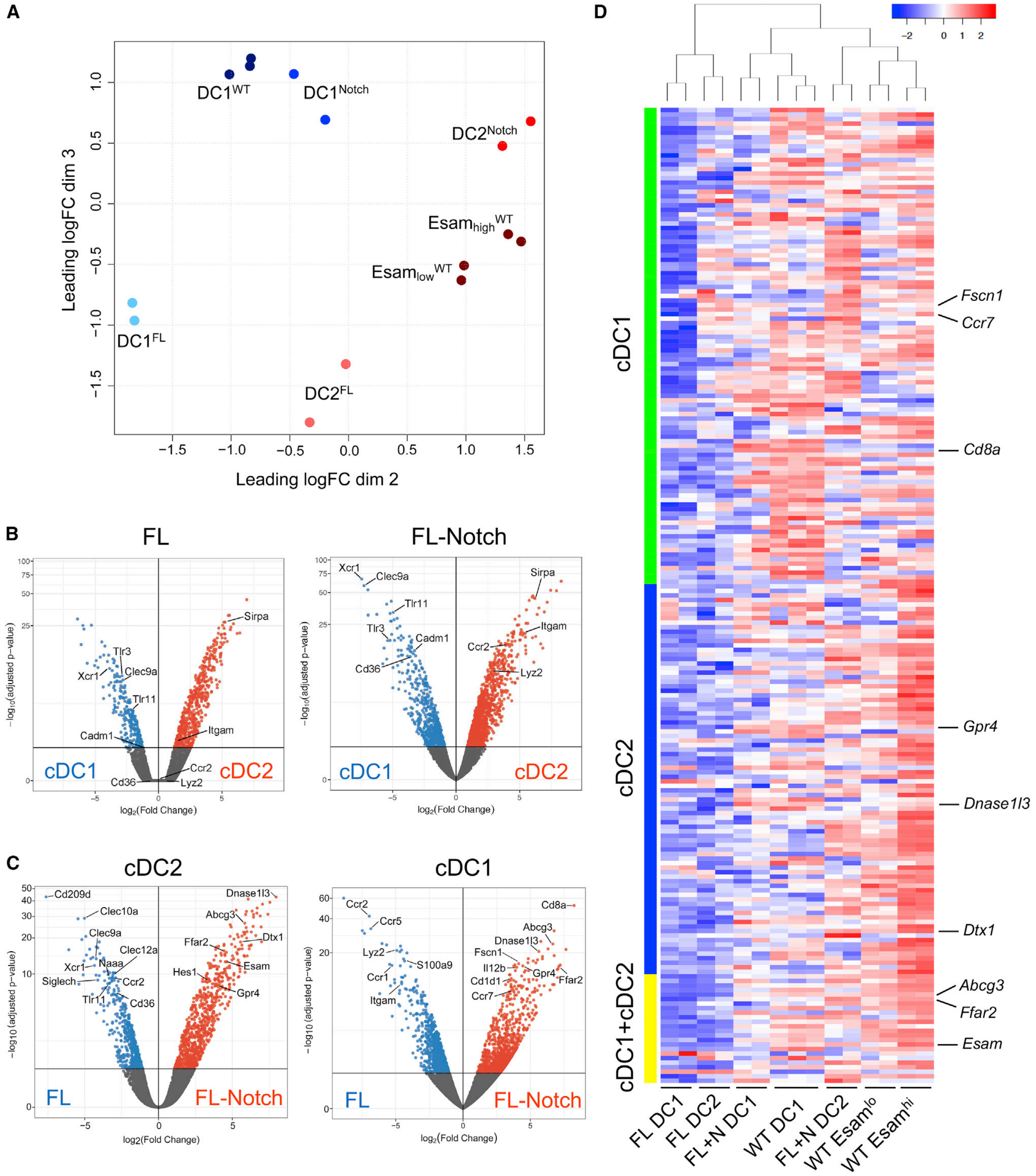

Figure 3. Notch Signaling Optimizes the Expression Profile of BM-Derived DCs

Duplicate samples of sorted DC subsets from FL and FL-Notch cultures of primary BM were analyzed by RNA-seq.

(A) Multidimensionality scaling (MDS) analysis of RNA-seq profiles of DC subsets derived from FL cultures ($DC1^{FL}$, $DC2^{FL}$), FL+Notch cultures ($DC1^{Notch}$, $DC2^{Notch}$), and primary splenic DC subsets from WT mice ($DC1^{WT}$, $Esam_{high}^{WT}$, and $Esam_{low}^{WT}$ DC2s). All samples are plotted on the second and third dimension of MDS.

(B) Pairwise comparison of RNA-seq profiles of cDC1 versus cDC2 subsets from the indicated culture conditions. Shown are volcano plots of individual genes, with select subset-specific marker genes highlighted.

(legend continued on next page)

2013), including canonical Notch targets (*Dtx1* and *Hes1*), characteristic markers (*Esam*), and DC-specific Notch target genes (*Dnase1l3*, *Abcg3*, *Ffar2*, and *Gpr4*). The downregulated genes included markers of Esam^{lo} cDC2 (*Clec12a*) and multiple genes associated with other DC subsets, including pDC (*Siglech*, *Clec10a*, and *CD209d*) and cDC1 (*Xcr1*, *Clec9a*, *CD36*, *Naaa*, and *Tlr11*). In the pairwise comparison of cDC1, the top upregulated gene was *CD8a*; other genes involved in cDC1 function were upregulated (*Il12b* and *Cd1d1*), as were DC-specific NOTCH2 targets (*Dnase1l3*, *Abcg3*, *Ffar2*, and *Gpr4*). Of particular interest was the induction of genes controlling DC migration, including the actin-bundling protein *Fscn1* (Yamashiro, 2012) and *Ccr7*, a receptor that guides DC migration from tissues to lymphoid organs (Worbs et al., 2017). Conversely, downregulated genes included cDC2 and/or myeloid markers (*Lyz2*, *S100a9*, and *Itgam*) and chemokine receptors that mediate DC migration from blood (*Ccr1*, *Ccr2*, and *Ccr5*). Together with the phenotypic analysis, these data show that Notch signaling optimizes DC subset-specific gene expression and drives their differentiation toward their *in vivo* counterparts.

Finally, we tested whether induced Notch signaling in culture recapitulates the Notch-dependent gene expression program of primary splenic DCs. We compiled genes that were differentially expressed in NOTCH2-deficient splenic cDC1, cDC2, or both subsets (Satpathy et al., 2013) and analyzed their expression in culture-derived and primary cDC (Table S3). The expression of both NOTCH2-induced genes (Figure 3D) and NOTCH2-repressed genes (Figure S3B) faithfully clustered cDC1$^{FL\text{-}Notch}$ and cDC2$^{FL\text{-}Notch}$ with their primary counterparts, whereas cDC1^{FL} and cDC2^{FL} clustered separately from primary cDCs. Both subsets showed upregulation of subset-specific and common Notch target genes, including the abovementioned *Dnase1l3*, *Abcg3*, *Ffar2*, *Gpr4*, *Esam*, and *Dtx1* (Figure 3D). Notably, cDC1-enriched Notch target genes included *CD8a*, *Fscn1*, and *Ccr7*, suggesting that their induction in cDC1$^{FL\text{-}Notch}$ reflects their natural regulation by Notch. Conversely, downregulated genes repressed by Notch included progenitor genes (e.g., *CD34* and *Cx3cr1*), subset-inappropriate genes (e.g., *Tlr4* in cDC1 and *CD36* in cDC2), and chemokine receptor *Ccr2* (Figure S3B). Thus, OP9-DL1 co-cultures facilitate cDC differentiation by recapitulating the physiological Notch-dependent gene expression program of primary cDCs.

Notch Signaling Optimizes Migratory Properties of *In Vitro*-Derived DCs

We examined the distinct functional properties of cDC1s from FL and FL-Notch cultures *in vitro*. Despite the increased baseline expression of *Il12b* (Figure 3C), cDC1$^{FL\text{-}Notch}$ did not show an enhanced interleukin-12 (IL-12) response to the TLR11 ligand profilin (data not shown). Cross-presentation of exogenous protein Ag to CD8$^+$ T cells is a hallmark property of cDC1 (den Haan et al., 2000; Hildner et al., 2008). To measure Ag cross-presentation *in vitro*, we pulsed DCs from either culture method with ovalbumin (OVA), washed and incubated them with H-2K^b-OVA peptide-specific T cell receptor (TCR) transgenic OT-I CD8$^+$ T cells, and measured OT-I proliferation by the dilution of the cell tracer dye carboxyfluorescein succinimidyl ester (CFSE). Total DCs and enriched cDC1s from both culture types induced comparably strong OT-I proliferation at a 1:1 DC:T cell ratio (data not shown). At lower DC:T cell ratios (1:5–1:10), total DCs and enriched cDC1s from FL-Notch cultures induced more extensive CFSE dilution in T cells (Figures 4A and 4B). Thus, Notch signaling is not strictly required for the cross-presenting capacity of cDC1 but facilitates T cell cross-priming under limiting conditions *in vitro*.

Comparison of DC^{FL} and DC$^{FL\text{-}Notch}$ expression profiles identified regulators of cell migration among the most differentially expressed genes (Figures 3C and 3D). We therefore analyzed the expression of chemokines and their receptors in culture-derived and primary DCs (Table S4). Unsupervised clustering by chemokine expression did not group DC$^{FL\text{-}Notch}$ with primary DCs (Figure S3C). Nevertheless, several chemokines were induced in cDC1$^{FL\text{-}Notch}$ compared with cDC1^{FL}, including *Cxcl9* (Figure S3D). Notably, CXCL9 is preferentially expressed in primary cDC1s (Figure S3D) and plays a major role in cDC1-mediated priming of tumor-specific T cell responses (de Mingo Pulido et al., 2018; Spranger et al., 2017). The expression of chemokine receptors separated DC^{FL} from other samples but clustered DC$^{FL\text{-}Notch}$ with their respective splenic DC counterparts (Figure 4C). Furthermore, cDC1$^{FL\text{-}Notch}$ showed a profound downregulation of *Ccr1*, *Ccr2*, and *Ccr5* and induction of *Ccr7*, aligning the expression of these receptors with primary cDC1 (Figure 4D). The induction of *Ccr7* in cDC1$^{FL\text{-}Notch}$ was particularly notable given its very low levels in cDC1^{FL}. Cell surface staining confirmed the induction of CCR7 expression on DC$^{FL\text{-}Notch}$ (Figure 4E). Accordingly, transwell migration assays showed that all DC^{FL} preferentially migrated toward the CCR2 ligand CCL2, with lower migration toward the CCR1 and CCR5 ligand CCL5 and CCR7 ligand CCL19 (Figure 4F). In contrast, DC$^{FL\text{-}Notch}$ failed to migrate toward CCL2 or CCL5, but showed increased migration toward CCL19, with the difference being particularly notable in the cDC1 subset (Figure 4F). These data suggest that Notch signaling induces a more physiological pattern of chemokine receptor expression and migration in cultured DCs, specifically favoring CCR7-dependent over CCR2- and CCR5-dependent migration.

Notch Signaling Facilitates cDC1-Mediated T Cell Cross-Priming and Antitumor Vaccination

Given the optimized cross-presenting and migratory properties of DC$^{FL\text{-}Notch}$ *in vitro*, we tested the ability of these cells to cross-prime T cell responses *in vivo*. DCs from FL or FL-Notch cultures were pulsed with OVA, and either total DCs or enriched CD24$^+$ cDC1s (Figure S4A) were transferred

(C) Pairwise comparison of RNA-seq profiles of the indicated DC subsets generated in FL versus FL-Notch cultures. Shown are volcano plots of individual genes, with select highly differentially expressed genes highlighted.

(D) Heatmap of Notch-dependent gene expression in cultured and primary DC subsets. Samples (labeled as in A) were hierarchically clustered by the expression of genes that were downregulated preferentially in *Notch2*-deficient cDC1s, cDC2s, or both cDC1s and cDC2s. Select genes are highlighted; the color scale represents the row *Z* score.

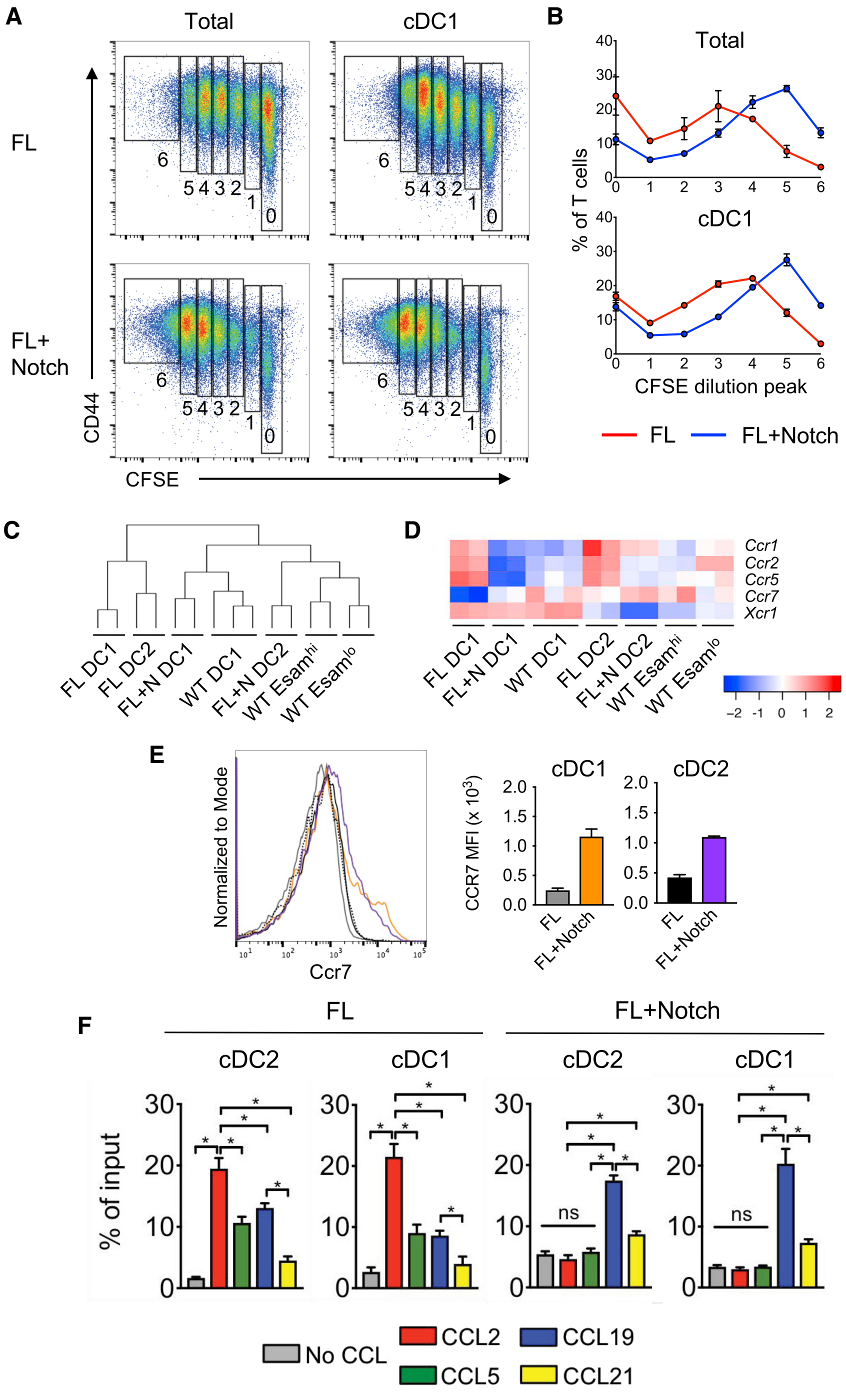

Figure 4. Notch Facilitates CCR7-Dependent Migration of DCs

DCs were generated by culturing murine BM cells in the presence of FLT3L alone (FL) or FLT3L with OP9 cells expressing the Notch ligand DL1 (FL+Notch).

(A and B) Cross-priming of $CD8^+$ T cells *in vitro*. FL or FL-Notch cultures on day 7 were incubated with OVA, and either total cultures or enriched cDC1s were incubated with CFSE-labeled OT-I cells at a 1:10 ratio for 3 days.

(A) The levels of CFSE versus the activation marker CD44 in gated $CD8^+$ T cells from cultures with the indicated DCs, with the individual peaks of CFSE dilution highlighted. No CFSE dilution was observed in the absence of OVA (data not shown).

(B) The fraction of T cells in each peak (mean of 4 parallel cultures ± S.D., representative of 2 experiments).

(C) Unsupervised clustering of cultured and primary splenic DCs by the expression of chemokine receptors (Table S4). Shown is the clustering dendrogram with individual replicates of the indicated samples.

(D) Heatmap of select chemokine receptor expression in cultured and primary splenic DCs as determined by RNA-seq. The color scale represents the row *Z* score.

(E) The expression of CCR7 on the surface of culture-derived DCs. Shown is a representative staining profile and averaged mean fluorescence intensity (MFI) of CCR7 on gated $CD11b^+$ cDC2s or $CD24^+$ cDC1s. Data represent mean ± range of 2 cultures.

(F) DC migration *in vitro* in a transwell assay. Total DCs from FL or FL+Notch cultures were seeded in top chambers with the indicated recombinant chemokine in the bottom chamber and allowed to migrate for 3 hr. Shown is the fraction of each DC subset that migrated into the bottom chamber. Data represent mean ± SD of 4 parallel transwell cultures, representative of 3 experiments.

Statistical significance: $^*p < 0.05$.

into wild-type syngeneic H-$2K^b$ recipients. One week later, OVA-specific endogenous $CD8^+$ T cells were detected with an H-$2K^b$-OVA peptide (SIINFEKL) tetramer. We found that up to 10^6 cells/animal of DC^{FL} failed to elicit OVA-specific T cells in the spleen (Figure S4B) and in the peripheral blood (Figures 5A and 5B). In contrast, $DC^{FL\text{-}Notch}$ induced robust T cell responses even at lower doses ($0.25–0.5 \times 10^6$ cells/animal) (Figure S4B; Figures 5A and 5B). Furthermore, a major improvement in T cell priming was also observed when the same experiments were done with enriched $cDC1^{FL\text{-}Notch}$ (Figures 5A and 5C). The comparison of total DCs to cDC1s in these experiments was confounded by the cDC1 isolation procedure and by potential saturation of the T cell response. Nevertheless, it is notable that neither total DC^{FL} nor enriched $cDC1^{FL}$ could cross-prime at any dose, whereas total $DC^{FL\text{-}Notch}$ and $cDC1^{FL\text{-}Notch}$ were comparably efficient.

To test the consequences of differential T cell priming, recipient mice were challenged with the OVA-expressing syngeneic melanoma cell line B16 (B16-OVA). After intravenous (i.v.) retroorbital administration of 2.5×10^5 B16-OVA cells, all untreated mice became moribund with respiratory distress within ~2 weeks and had to be sacrificed (Figure 5D). Histological

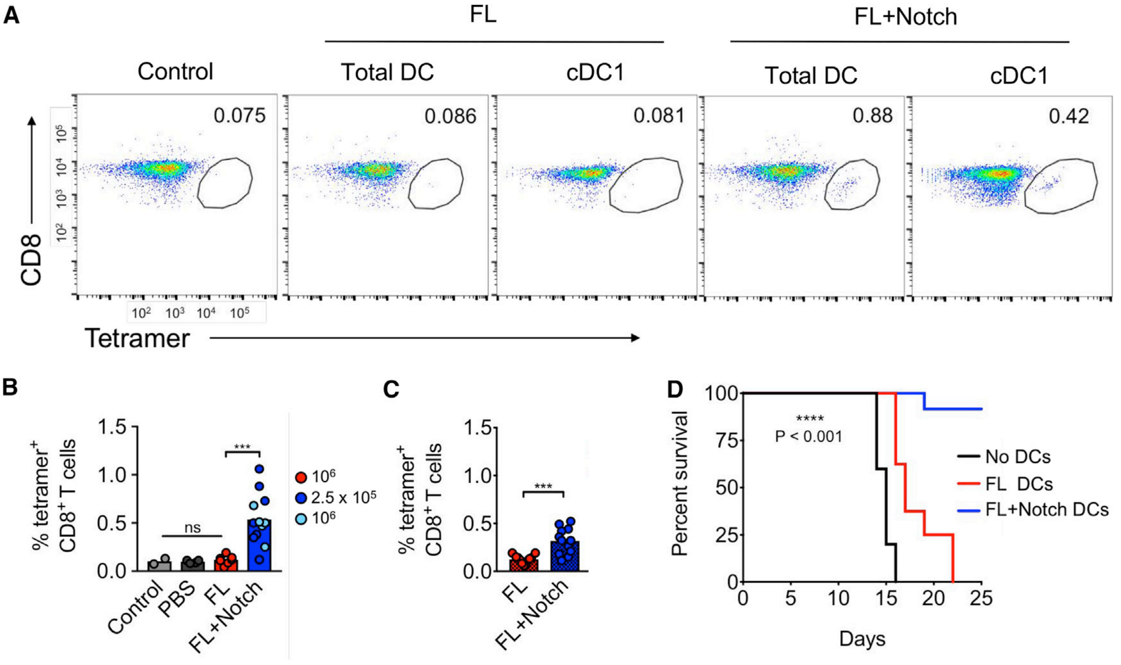

Figure 5. Notch Facilitates cDC1-Mediated T Cell Cross-Priming *In Vivo*

DCs from FL or FL+Notch cultures of primary murine BM were incubated with OVA, and total DCs or enriched cDC1s were injected i.v. into naive WT syngeneic recipient mice. The priming of endogenous OVA peptide-specific $CD8^+$ T cells was determined 7 days later by staining of PB leukocytes with H-$2K^b$-OVA peptide tetramer.

(A) Representative staining profiles of gated $CD44^+$ $TCR\beta^+$-activated T cells in the PB, with the $CD8^+$ $tetramer^+$ cells highlighted.

(B) The fraction of OVA-specific T cells among total $CD44^+$ $TCR\beta^+$ $CD8^+$ cells after vaccination with the indicated numbers of total DCs. Mice that received no injection (control) or a mock PBS injection are shown as well. Data points represent values from individual mice pooled from 2 experiments; bars represent mean.

(C) The fraction of OVA-specific T cells after vaccination with 3×10^5 enriched cDC1s. Data points represent values from individual mice pooled from 3 experiments; bars represent mean.

(D) Kaplan-Meier survival plot of animals that were vaccinated with OVA-pulsed total DCs and subsequently challenged with the OVA-expressing melanoma cell line.

Statistical significance: ****$p < 0.0001$, ***$p < 0.001$.

analysis of the lungs showed disseminated infiltration of the alveolar space by B16 cells, likely causing respiratory failure (Figure S4C). A similar mortality was observed in all mice vaccinated with OVA-pulsed total DC^{FL} (Figure 5D). In contrast, vaccination with OVA-pulsed total $DC^{FL\text{-}Notch}$ conferred near-complete protection against B16-OVA challenge (Figure 5D). The single $DC^{FL\text{-}Notch}$ recipient that succumbed to the tumor harbored a low fraction (<0.2%) of OVA-specific T cells, confirming the dependence of protection on T cell priming. We also performed these experiments injecting B16-OVA cells i.v. through the tail vein; this route of administration yielded the characteristic focal growth of melanoma cells in the lungs. Again, vaccination with OVA-pulsed DC^{FL} had no effect, whereas vaccination with $DC^{FL\text{-}Notch}$ strongly reduced the growth of melanoma foci (Figure S4D). We conclude that FL-Notch culture generates cDC1s with a superior capacity for T cell cross-priming and antitumor vaccination.

Notch Signaling Facilitates Development of cDC1s from Human Hematopoietic Progenitors

The potential therapeutic properties of cDC1s, but their rarity *in vivo*, led us to explore *in vitro* differentiation approaches in humans. Culture of BM $CD34^+$ progenitors in liquid medium supplemented with the cytokines FLT3L, stem cell factor (SCF), and GM-CSF (FSGM) generated only $CD14^+$ and $CD1c^+$ monocyte-like or cDC2-like cells (Figure 6A). Addition of an OP9 feeder layer supported the simultaneous generation of all three DC subsets with a low yield of 0.4 cDC1s per input $CD34^+$ progenitor (Figure 6B). Addition of a Notch signal by co-culture with OP9-DL1 cells resulted in selective expansion of cDC1s, increasing the cDC1 output ~11-fold (4.4 cDC1s per input progenitor) on day 14 of culture. A lower expansion of cDC1s (2.8 cDC1s/progenitor) was seen on exposure of progenitors to OP9-DL4 under similar culture conditions (Figures 6A and 6B). cDC1 expansion was dependent on a continuous Notch signal because withholding or withdrawing Notch ligand for the first or last 7 days, respectively, decreased the effect (Figure S5A).

Culture-generated cDC1s expressed the human cDC1-specific markers CD141 and CLEC9A at levels comparable with or higher than *ex vivo* blood cDC1s (Figure S5B). However, they expressed low levels of CD11c and high levels of CD1c, differing from peripheral blood (PB) cDC1s but resembling the phenotype of peripheral tissue cDC1s (Figure S5B). Next we performed a gene expression analysis on the NanoString nCounter platform

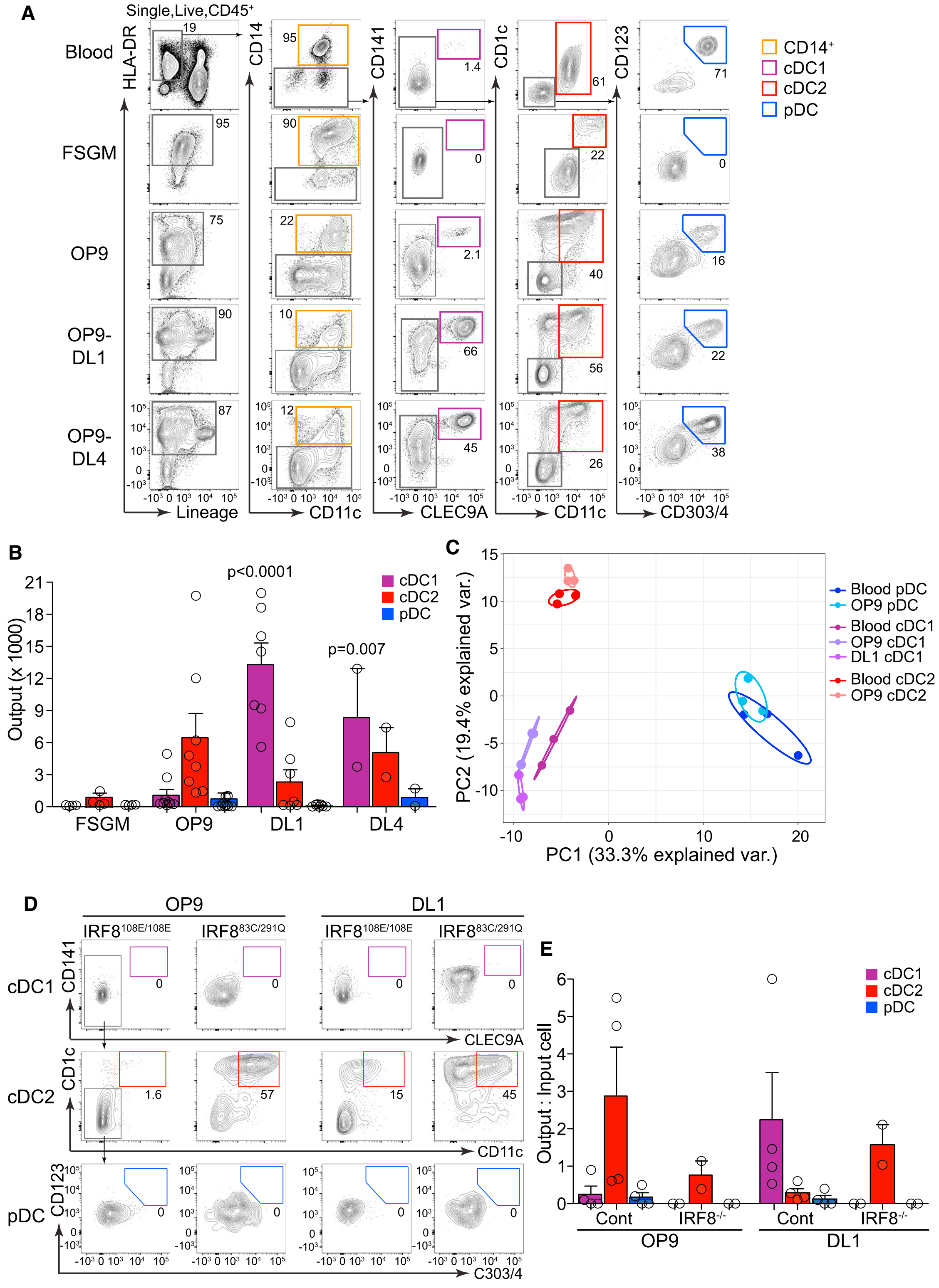

Figure 6. Notch Facilitates cDC1 Development from Human BM Progenitors

(A and B) Sorted CD34+ stem and progenitor cells purified from human BM were cultured for 2 weeks in the presence of a FLT3L-containing cytokine mix (FSGM) or on monolayers of control OP9, OP9-DL1, or OP9-DL4 cells.

(legend continued on next page)

running the Human Immunology v2 panel (594 genes) with the addition of 30 DC-specific genes (Table S5). Expression profiles were compared after removal of the genes with low expression levels and of the genes that were differentially expressed between all culture and all *ex vivo* subsets (the cell culture signature). By subsequent principal-component analysis based on 339 genes, the first component separated pDCs from cDCs and the second cDC1s from cDC2s (Figure 6C). Notably, OP9-DL1-differentiated cDC1s grouped closely with OP9-derived cDC1s and with primary cDC1s from the PB. Accordingly, genes encoding subset-specific transcription factors, surface markers, and TLRs were expressed faithfully, including *CLEC9A*, *XCR1*, *TLR3*, *IRF8*, and *BATF3* in cDC1s (Figure S5C). The expression of chemokine receptors was similarly segregated by subset (Figure S5D), although a higher expression of CCR7 in OP9-derived cDC1s and cDC2s was noted. In contrast, the expression of chemokines segregated OP9-derived cDC1s and cDC2s, which showed higher levels of *CXCL10*, *CXCL12*, and *CCL22* than either DL1-derived DCs or primary DCs (Figure S5E). Thus, unlike in the mouse system, OP9-DL1 does not appear to affect the overall expression profile or the expression of migration regulators in cDC1s. Nevertheless, we conclude that DL1-induced Notch signaling greatly increases the number of differentiated cDC1s with the appropriate expression profile.

Biallelic *IRF8* mutation in human abrogates pDC, cDC1, and cDC2 development *in vivo* (Bigley et al., 2017; Hambleton et al., 2011). To interrogate the IRF8 requirement for *in vitro* Notch-induced cDC1 expansion, IRF8$^{108E/108E}$ and IRF8$^{83C/291Q}$ CD34^{+} progenitors were co-cultured with either OP9 or OP9-DL1. cDC1s and pDCs failed to emerge under either condition, whereas some cDC2 generation was observed (Figures 6D and 6E). Thus, Notch signaling from DL1 specifically facilitates the development of human cDC1s with the appropriate phenotype, expression profile, and genetic requirements.

Notch-Driven Differentiation Results in Functional Human cDC1s

To interrogate the functional capacity of *in vitro*-generated DCs, we examined cytokine production in response to TLR agonists. PB mononuclear cells or *in vitro*-derived DCs were exposed to a cocktail of TLR agonists (CL075, CpG, lipopolysaccharide [LPS], and poly-I:C), and subset-specific cytokine production was assessed by intracellular flow cytometry (Figures 7A and 7B). Tumor necrosis factor (TNF) production by cDC1s was significantly increased in OP9-DL1-derived compared with OP9-derived cells and comparable with blood cDC1s. No significant increase in IL-12 production was observed in cDC1s or cDC2s generated *in vitro*, but a decrease in both TNF and interferon α (IFN-α) was observed in DL1-derived pDCs (Figure 7B).

Culture-derived cDC1s showed appropriate migration in a transwell assay toward the XCR1 ligand XCL1 as well as weaker migration toward CCR2, CCR5, and CCR7 ligands; no major differences between OP9- and OP9-DL1-derived DCs were noted (data not shown). To assess their T cell-stimulatory capacity, cDC1s and cDC2s from the PB or from *in vitro* cultures were sorted and co-cultured with allogeneic T cells. The proportion of CD4^{+} or CD8^{+} T cells that underwent division was determined by CFSE dilution (Figure 7C). It should be noted that no differences were expected between CD4^{+} and CD8^{+} T cells in this assay, which measures direct T cell activation rather than cross-priming. cDC1s generated on OP9-DL1 stimulated proliferation in a significantly greater proportion of both CD4^{+} and CD8^{+} T cells compared with those generated on OP9; this effect was not observed in cDC2s (Figure 7D). We conclude that Notch signaling induces the development of functionally intact cDC1s, which show a significant improvement in their *in vitro* T cell priming capacity. Together with the drastic increase of cDC1 yield, these data underscore the improvement of cDC1 generation in Notch-driven cultures.

DISCUSSION

We describe an *in vitro* method of DC generation based on the combination of two cell-extrinsic signals, FLT3L and Notch. FLT3L is the key cytokine for the DC lineage and alone is sufficient to drive DC development and subset specification from murine hematopoietic progenitors. Murine FLT3L-derived cDCs harbor a population of IRF8-dependent cDC1-like cells (cDC1^{FL}) that manifest functional hallmarks of cDC1s, such as IL-12 production and T cell cross-priming *in vitro* (Naik et al., 2005). However, these cells have an abnormal CD11b^{+} CD8α^{-} Dec205^{-} CD103^{-} phenotype, fail to migrate toward CCR7, and are shown here to have a poor capacity for T cell cross-priming *in vivo*. A combination of FLT3L with the cytokine GM-CSF in a two-step culture selectively expands cDC1-like cells and confers CD103 expression, yielding so-called iCD103-DCs (Mayer et al., 2014). However, iCD103-DCs have the same aberrant CD11b^{+} CD8α^{-} Dec205^{low} phenotype and lack CCR7 expression unless stimulated with TLR ligands. Moreover, their expression profile and functional properties were never directly compared with those of cDC1^{FL}, and their T cell priming capacity *in vivo* was tested only after TLR-induced activation (Mayer et al., 2014). Thus, apart from the expected induction of CD103 (Zhan et al., 2011), GM-CSF does not appear to improve the quality of FLT3L-derived cDCs. Conversely, stromal cells expressing the

(A) Representative staining profiles highlighting the indicated DC subsets in the resulting cultures; primary DCs from the PB are included for comparison. Numbers represent the percentage of gated cells.
(B) Absolute numbers of DC subsets generated in a 0.2-mL culture standardized to 3,000 CD34^{+} progenitor cell input. Data points represent values in BM cultures from different donors (n = 8 for OP9, 7 for OP9-DL1, and 2 for OP9-DL4); bars represent mean with SEM. The indicated p values were derived by unpaired two-tailed t test. The data represent the absolute number of DC subsets per input progenitor cell.
(C) Comparison of expression profiles of culture-derived and primary DC subsets based on the Nanostring nCounter analysis. Shown is the principal-component analysis of the indicated triplicate samples after removal of the "culture signature" derived by pairwise comparison of all culture-generated versus *ex vivo* cells.
(D and E) Representative staining profiles (D) and cell yields (E) of the cultures of BM from the two patients with biallelic *IRF8* mutations (IRF8$^{108E/108E}$ or IRF8$^{83C/291Q}$). The experiments were done as in (A) and (B).

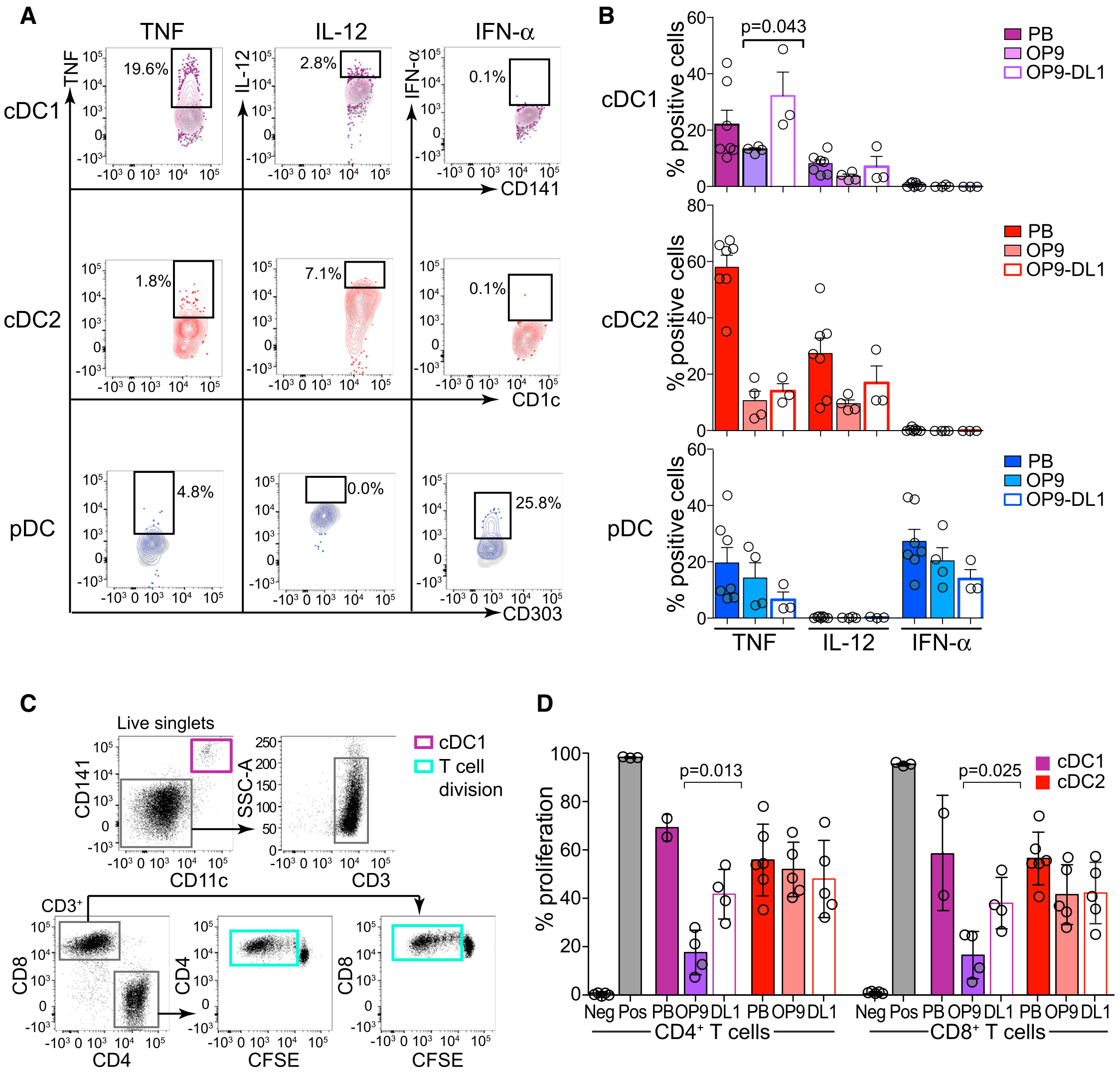

Figure 7. Notch-Driven Differentiation Yields Functional Human cDC1s

DCs generated from CD34$^+$ progenitors in cultures with OP9 or OP9-DL1 were analyzed in parallel to primary DCs from PB.

(A and B) Cytokine production by DCs stimulated for 14 hr with a cocktail of TLR agonists (poly-I:C, LPS, CL075, and CpG).

(A) Representative flow cytometric analysis of intracellular cytokine production (TNF, IL-12, and IFN-α) in the indicated gated DC subsets from OP9-DL1 cultures. Grey contours represent unstimulated cells; numbers represent the cytokine-positive fraction.

(B) Proportion of cytokine-positive cDC1s (purple), cDC2s (red), or pDCs (blue) generated from CD34$^+$ progenitors in culture with OP9 (n = 4) or OP9-DL1 (n = 3) cells compared with PB primary (n = 7) cells following TLR stimulation. Circles, histograms, and bars represent individual experiments, mean, and SEM, respectively; p values are indicated.

(C and D) T cell stimulation by DCs cultured with sorted allogeneic blood CD3$^+$ T cells.

(C) Representative flow cytometric analysis of T cell proliferation in response to culture with DCs. The data show output of a T cell and cDC1 (generated with OP9-DL1) culture. CD11c$^+$ CD141$^+$ cDC1s could be identified (purple gate) and gated out. CD3$^+$ T cells were subdivided by CD8 and CD4 expression. Cell division was indicated by CFSE dilution (turquoise gate).

(D) Proportion of CD4$^+$ or CD8$^+$ T cells that underwent division (CFSE dilution) in culture with cDC1 (purple) or cDC2 (red) isolated from PB or generated in culture with OP9 or OP9-DL1 (DL1) cells. T cells cultured alone or with beads coated with anti-CD3 plus anti-CD28 were used as negative (Neg) and positive (Pos) controls, respectively. Responses to blood DCs were generated from 2–3 DC donors and 3 T cell donors (2–6 independent experiments). Responses to cultured DCs were generated from 2 BM donors combined with 3 T cell donors (4–6 independent experiments). Each circle represents an independent experiment (mean of 1–3 technical replicates). Histograms and bars represent mean and SEM, respectively. The p values were derived from unpaired two-tailed Student's t test.

Notch ligand DL1 were reported to improve DC differentiation in GM-CSF cultures, but these do not generate any cDC1 output (Cheng et al., 2007).

Here, we show that the introduction of the Notch ligand DL1 into FLT3L-driven culture using the OP9-DL1 stromal cell line (Schmitt et al., 2004) yields the two main cDC subsets, including

bona fide CD11b^{-} CD8α^{+} Dec205^{+} CD103^{+} cDC1s. Indeed, cDC1s and cDC2s became more distinct by key surface markers and by global expression profile, at the same time better resembling their primary counterparts in the spleen. Notably, cDC2s showed higher similarity to the Esam^{hi} subset of splenic cDC2s, which manifest a superior ability to prime T cells (Lewis et al., 2011). Most importantly, the cDC1 subset manifested the appropriate surface phenotype and expression profile and an improved ability to cross-prime CD8^{+} T cell responses. The latter underscores a dramatic improvement in the quality of resulting cDC1s, even as the fraction and numbers of cDC1s were not increased. Indeed, Notch-derived cDC1s dramatically outperformed conventional cDC1-like cells in T cell priming *in vivo*, even at much lower numbers. Moreover, the potential limitation of cell numbers can be overcome by our system's application to immortalized DC progenitors, which can be scaled up indefinitely.

The improved functionality of Notch-derived cDC1s does not appear to reflect an effect on Ag cross-presentation capacity per se because cDC1-like cells from FL cultures could cross-present *in vitro*, as described previously (Naik et al., 2005). Instead, it is likely due to an enhanced capacity to recruit T cells (e.g., through the elevated expression of CXCL9) and/or to migrate toward appropriate locations in lymphoid organs. These functions are less important when DCs and T cells interact in the artificial context of *in vitro* cultures (except at lower DC:T cell ratios) but are critical for T cell priming *in vivo*. FL-Notch co-cultures switched the chemokine receptor expression pattern of the resulting DCs from CCR1, CCR2, and CCR5 toward CCR7, recapitulating that of DCs in lymphoid organs. This switch facilitated the migration toward CCR7 ligands, the process that guides DCs into T cell zones and is essential for T cell priming *in vivo* (Worbs et al., 2017). Collectively, Notch signaling increases the similarity of *in vitro*-generated DCs to their primary counterparts in the lymphoid organs, optimizing the functional properties of cDC1s.

Notch signaling is important for the differentiation of cDC2s in the spleen and intestine; indeed, these cells express Notch target genes such as *Hes1* and *Dtx1*, are located in DL1-expressing splenic niches, and depend on NOTCH2 and the canonical RBPJ-mediated signaling downstream of it (Caton et al., 2007; Lewis et al., 2011). In contrast, cDC1s express few or no Notch target genes and are unaffected by DC-specific loss of *Rbpj* (Caton et al., 2007); however, *Notch2* deletion impairs their phenotype and expression profile (Lewis et al., 2011; Satpathy et al., 2013). Consistent with the latter observation, our results identify the DL1-NOTCH2 axis as a signal that is missing in conventional FLT3L cultures and whose induction can drive differentiation into authentic functional cDC1s. To reconcile these data, we propose that DL1-NOTCH2 signaling is an important extrinsic signal for cDCs in the spleen (and possibly other lymphoid organs) that acts on committed cDC progenitors to facilitate cDC1 and cDC2 subset specification and terminal differentiation. This signal then stays "on" in Esam^{hi} cDC2, which remain in contact with DL1-expressing stroma and are fully dependent on DL1 signaling through NOTCH2-RBPJ for their survival. In contrast, the DL1-NOTCH2 signal may be turned "off" as cDC1s migrate throughout the spleen and lose Notch target gene expression and NOTCH2-RBPJ dependence. Overall, our studies emphasize the critical role of Notch signaling in the functional differentiation of both cDC subsets and demonstrate its utility to facilitate cDC generation *in vitro*.

FLT3L alone appears to be insufficient to drive DC development from human hematopoietic progenitors and has to be supplemented with other cytokines (Balan et al., 2014; Poulin et al., 2010; Proietto et al., 2012) or stromal cell lines, including MS5 alone (Lee et al., 2015) or a mixture of MS5 and OP9 (Lee et al., 2017). These conditions can generate mature CD141^{+} cDC1s with the appropriate phenotype, expression profile, and *in vitro* functionality. However, the fraction and absolute numbers of the resulting cDC1s are low in all cases, hampering their detailed functional characterization and practical use. This situation appears to be different from that in the mouse, where FLT3L alone can drive the development of abundant but not fully mature cDC1-like cells. It was recently reported that co-culture of human thymic progenitors with OP9-DL1 blocked the emergence of DC progenitors but increased the yield of cDCs from the latter (Martín-Gayo et al., 2017); neither the net effect on DC development nor the resulting DC phenotypes were investigated. We report that, although co-culture of human BM progenitors with OP9 generates all DC subsets, addition of the Notch ligand DL1 resulted in a more than 10-fold increase in cDC1 output per progenitor cell. The resulting cDC1s aligned closely with their *ex vivo* counterparts by gene expression analysis, were strictly IRF8-dependent, and showed increased T cell-stimulatory capacity. In contrast to mouse BM cultures, DL1 was required continuously from the beginning of the culture; furthermore, unlike in the mouse system, no major effects of DL1 on the phenotype, expression profile, or migration of the resulting cDC1s were noted. This may reflect different comparators for OP9-DL1 cultures ("FLT3L only" in the mouse and "FLT3L with other cytokines and OP9" in the human); differences in the effect of murine OP9 cells and their products (cytokines, adhesion molecules) on murine versus human cells; and/or biological differences between the two species. In each case, however, Notch signaling appeared to solve a major hurdle to cDC1 differentiation; i.e., it improve the suboptimal expression profile and functionality in the mouse and increase low numbers in humans. Collectively, these results emphasize the conserved positive effect of Notch signaling on *in vitro* cDC1 differentiation.

The long-standing idea of DC-based vaccination against tumors (Palucka and Banchereau, 2013) is now being actively pursued in human patient studies, especially in combination with other immunotherapies (Garg et al., 2017). cDC1s appear to play a particularly important role in antitumor responses through their efficient cross-priming of tumor-specific cytotoxic T cells (Bottcher et al., 2018; Roberts et al., 2016; Salmon et al., 2016; Spranger et al., 2017). The utility of cDC1s for antitumor vaccination is further illustrated by our results in the mouse, in which Notch-derived cDC1s protected against tumor challenge whereas larger numbers of total FL-derived DCs did not. The utility of human cDC1s for antitumor vaccination so far has been severely limited by their rarity *in vivo* and low yield *in vitro*. Our system overcomes this hurdle by increasing the output of human cDC1s by an order of magnitude, facilitating their potential use for vaccination. Notably, Notch-derived cDC1s are functional *in vivo* without prior TLR-induced activation, an important

advantage for translational applications. Furthermore, the activation-independent functionality of Notch-derived DCs supports their potential application for tolerogenic DC vaccination; e.g., against autoimmune diseases. Collectively, the methods of *in vitro* DC generation described here should facilitate mechanistic and translational studies focused on the therapeutic potential of DCs.

STAR★METHODS

Detailed methods are provided in the online version of this paper and include the following:

- KEY RESOURCES TABLE
- CONTACT FOR REAGENT AND RESOURCE SHARING
- EXPERIMENTAL MODEL AND SUBJECT DETAILS
  - Human studies
  - Animals
  - Cell lines
- METHOD DETAILS
  - Methods: mouse
  - Methods: human
- QUANTIFICATION AND STATISTICAL ANALYSIS
- DATA AND SOFTWARE AVAILABILITY

SUPPLEMENTAL INFORMATION

Supplemental Information includes five figures and five tables and can be found with this article online at https://doi.org/10.1016/j.celrep.2018.05.068.

ACKNOWLEDGMENTS

We gratefully acknowledge the participation of healthy donors and patients. We thank Ioannis Aifantis, Stefan Feske, and their lab members for reagents and advice; Juan Carlos Zúñiga-Pflücker and the Sunnybrook Research Institute for OP9-DL1/4 cells; and Hans Hacker for HoxB8-FL cells. We acknowledge the Newcastle University Flow Cytometry Core Facility, the NYU Genome Technology Center (GTC), and NYU Applied Bioinformatics Facility Laboratories (ABL). This work has used computing resources at the NYU High Performance Computing Facility (HPCF). The GTC and ABL are shared resources partially supported by NIH grant P30CA016087 at the Laura and Isaac Perlmutter Cancer Center. This work was supported by NIH grants AI072571, AG049074, and AI115382 (to B.R.); AI100853 (to C.M.L.); and AI124661 (to M.E.K.); Wellcome Trust 101155/Z/13/Z (to V.B. and U.C.), CRUK C30484/A21025 (to M.C.), and the NIHR Newcastle Biomedical Research Centre (to V.B., U.C., and M.C.).

AUTHOR CONTRIBUTIONS

M.E.K., U.C., C.M.L., and K.L.L. performed and interpreted experiments. C.W.S., H.S., and M.M. provided reagents and expertise. A.K.-J., under A.T.'s supervision, and A.R. performed the bioinformatic analysis. M.C., V.B., and B.R. supervised the project. M.E.K., U.C., M.C., V.B., and B.R. wrote the manuscript with input from all coauthors.

DECLARATION OF INTERESTS

C.W.S. is an employee of Genentech, Inc.

Received: January 29, 2018
Revised: April 24, 2018
Accepted: May 18, 2018
Published: June 19, 2018

REFERENCES

Aliberti, J., Schulz, O., Pennington, D.J., Tsujimura, H., Reis e Sousa, C., Ozato, K., and Sher, A. (2003). Essential role for ICSBP in the in vivo development of murine CD8alpha + dendritic cells. Blood *101*, 305–310.

Anders, S., Pyl, P.T., and Huber, W. (2015). HTSeq–a Python framework to work with high-throughput sequencing data. Bioinformatics *31*, 166–169.

Balan, S., Ollion, V., Colletti, N., Chelbi, R., Montanana-Sanchis, F., Liu, H., Vu Manh, T.P., Sanchez, C., Savoret, J., Perrot, I., et al. (2014). Human XCR1+ dendritic cells derived in vitro from CD34+ progenitors closely resemble blood dendritic cells, including their adjuvant responsiveness, contrary to monocyte-derived dendritic cells. J. Immunol. *193*, 1622–1635.

Bigley, V., Maisuria, S., Cytlak, U., Jardine, L., Care, M.A., Green, K., Gunawan, M., Milne, P., Dickinson, R., Wiscombe, S., et al. (2017). Biallelic interferon regulatory factor 8 mutation: A complex immunodeficiency syndrome with dendritic cell deficiency, monocytopenia, and immune dysregulation. J. Allergy Clin. Immunol., S0091-6749(17)31736-0.

Bottcher, J.P., Bonavita, E., Chakravarty, P., Blees, H., Cabeza-Cabrerizo, M., Sammicheli, S., Rogers, N.C., Sahai, E., Zelenay, S., and Reis, E.S.C. (2018). NK Cells Stimulate Recruitment of cDC1 into the Tumor Microenvironment Promoting Cancer Immune Control. Cell *172*, 1022–1037.e14.

Caton, M.L., Smith-Raska, M.R., and Reizis, B. (2007). Notch-RBP-J signaling controls the homeostasis of CD8- dendritic cells in the spleen. J. Exp. Med. *204*, 1653–1664.

Cheng, P., Nefedova, Y., Corzo, C.A., and Gabrilovich, D.I. (2007). Regulation of dendritic-cell differentiation by bone marrow stroma via different Notch ligands. Blood *109*, 507–515.

de Mingo Pulido, A., Gardner, A., Hiebler, S., Soliman, H., Rugo, H.S., Krummel, M.F., Coussens, L.M., and Ruffell, B. (2018). TIM-3 Regulates CD103(+) Dendritic Cell Function and Response to Chemotherapy in Breast Cancer. Cancer cell *33*, 60–74.e6.

den Haan, J.M., Lehar, S.M., and Bevan, M.J. (2000). CD8(+) but not CD8(-) dendritic cells cross-prime cytotoxic T cells in vivo. J. Exp. Med. *192*, 1685–1696.

Dobin, A., Davis, C.A., Schlesinger, F., Drenkow, J., Zaleski, C., Jha, S., Batut, P., Chaisson, M., and Gingeras, T.R. (2013). STAR: ultrafast universal RNA-seq aligner. Bioinformatics *29*, 15–21.

Falo, L.D., Jr., Kovacsovics-Bankowski, M., Thompson, K., and Rock, K.L. (1995). Targeting antigen into the phagocytic pathway in vivo induces protective tumour immunity. Nat. Med. *1*, 649–653.

Fasnacht, N., Huang, H.Y., Koch, U., Favre, S., Auderset, F., Chai, Q., Onder, L., Kallert, S., Pinschewer, D.D., MacDonald, H.R., et al. (2014). Specific fibroblastic niches in secondary lymphoid organs orchestrate distinct Notch-regulated immune responses. J. Exp. Med. *211*, 2265–2279.

Feng, J., Wang, H., Shin, D.M., Masiuk, M., Qi, C.F., and Morse, H.C., 3rd. (2011). IFN regulatory factor 8 restricts the size of the marginal zone and follicular B cell pools. J. Immunol. *186*, 1458–1466.

Garg, A.D., Coulie, P.G., Van den Eynde, B.J., and Agostinis, P. (2017). Integrating Next-Generation Dendritic Cell Vaccines into the Current Cancer Immunotherapy Landscape. Trends Immunol. *38*, 577–593.

Grajkowska, L.T., Ceribelli, M., Lau, C.M., Warren, M.E., Tiniakou, I., Nakandakari Higa, S., Bunin, A., Haecker, H., Mirny, L.A., Staudt, L.M., and Reizis, B. (2017). Isoform-Specific Expression and Feedback Regulation of E Protein TCF4 Control Dendritic Cell Lineage Specification. Immunity *46*, 65–77.

Guilliams, M., Ginhoux, F., Jakubzick, C., Naik, S.H., Onai, N., Schraml, B.U., Segura, E., Tussiwand, R., and Yona, S. (2014). Dendritic cells, monocytes and macrophages: a unified nomenclature based on ontogeny. Nat. Rev. Immunol. *14*, 571–578.

Hambleton, S., Salem, S., Bustamante, J., Bigley, V., Boisson-Dupuis, S., Azevedo, J., Fortin, A., Haniffa, M., Ceron-Gutierrez, L., Bacon, C.M., et al. (2011). IRF8 mutations and human dendritic-cell immunodeficiency. N. Engl. J. Med. *365*, 127–138.

Haniffa, M., Bigley, V., and Collin, M. (2015). Human mononuclear phagocyte system reunited. Semin. Cell Dev. Biol. *41*, 59–69.

Helft, J., Böttcher, J., Chakravarty, P., Zelenay, S., Huotari, J., Schraml, B.U., Goubau, D., and Reis e Sousa, C. (2015). GM-CSF Mouse Bone Marrow Cultures Comprise a Heterogeneous Population of CD11c(+)MHCII(+) Macrophages and Dendritic Cells. Immunity *42*, 1197–1211.

Hildner, K., Edelson, B.T., Purtha, W.E., Diamond, M., Matsushita, H., Kohyama, M., Calderon, B., Schraml, B.U., Unanue, E.R., Diamond, M.S., et al. (2008). Batf3 deficiency reveals a critical role for CD8alpha+ dendritic cells in cytotoxic T cell immunity. Science *322*, 1097–1100.

Jackson, J.T., Hu, Y., Liu, R., Masson, F., D'Amico, A., Carotta, S., Xin, A., Camilleri, M.J., Mount, A.M., Kallies, A., et al. (2011). Id2 expression delineates differential checkpoints in the genetic program of CD8α+ and CD103+ dendritic cell lineages. EMBO J. *30*, 2690–2704.

Lau, C.M., Nish, S.A., Yogev, N., Waisman, A., Reiner, S.L., and Reizis, B. (2016). Leukemia-associated activating mutation of Flt3 expands dendritic cells and alters T cell responses. J. Exp. Med. *213*, 415–431.

Lee, J., Breton, G., Oliveira, T.Y., Zhou, Y.J., Aljoufi, A., Puhr, S., Cameron, M.J., Sékaly, R.P., Nussenzweig, M.C., and Liu, K. (2015). Restricted dendritic cell and monocyte progenitors in human cord blood and bone marrow. J. Exp. Med. *212*, 385–399.

Lee, J., Zhou, Y.J., Ma, W., Zhang, W., Aljoufi, A., Luh, T., Lucero, K., Liang, D., Thomsen, M., Bhagat, G., et al. (2017). Lineage specification of human dendritic cells is marked by IRF8 expression in hematopoietic stem cells and multipotent progenitors. Nat. Immunol. *18*, 877–888.

Lewis, K.L., Caton, M.L., Bogunovic, M., Greter, M., Grajkowska, L.T., Ng, D., Klinakis, A., Charo, I.F., Jung, S., Gommerman, J.L., et al. (2011). Notch2 receptor signaling controls functional differentiation of dendritic cells in the spleen and intestine. Immunity *35*, 780–791.

Love, M.I., Huber, W., and Anders, S. (2014). Moderated estimation of fold change and dispersion for RNA-seq data with DESeq2. Genome Biol. *15*, 550.

Mach, N., Gillessen, S., Wilson, S.B., Sheehan, C., Mihm, M., and Dranoff, G. (2000). Differences in dendritic cells stimulated in vivo by tumors engineered to secrete granulocyte-macrophage colony-stimulating factor or Flt3-ligand. Cancer Res. *60*, 3239–3246.

Martín-Gayo, E., González-García, S., García-León, M.J., Murcia-Ceballos, A., Alcain, J., García-Peydró, M., Allende, L., de Andrés, B., Gaspar, M.L., and Toribio, M.L. (2017). Spatially restricted JAG1-Notch signaling in human thymus provides suitable DC developmental niches. J. Exp. Med. *214*, 3361–3379.

Mayer, C.T., Ghorbani, P., Nandan, A., Dudek, M., Arnold-Schrauf, C., Hesse, C., Berod, L., Stüve, P., Puttur, F., Merad, M., and Sparwasser, T. (2014). Selective and efficient generation of functional Batf3-dependent CD103+ dendritic cells from mouse bone marrow. Blood *124*, 3081–3091.

Merad, M., Sathe, P., Helft, J., Miller, J., and Mortha, A. (2013). The dendritic cell lineage: ontogeny and function of dendritic cells and their subsets in the steady state and the inflamed setting. Annu. Rev. Immunol. *31*, 563–604.

Mildner, A., and Jung, S. (2014). Development and function of dendritic cell subsets. Immunity *40*, 642–656.

Mohtashami, M., Zarin, P., and Zúñiga-Pflücker, J.C. (2016). Induction of T Cell Development In Vitro by Delta-Like (Dll)-Expressing Stromal Cells. Methods Mol. Biol. *1323*, 159–167.

Murphy, T.L., Grajales-Reyes, G.E., Wu, X., Tussiwand, R., Briseño, C.G., Iwata, A., Kretzer, N.M., Durai, V., and Murphy, K.M. (2016). Transcriptional Control of Dendritic Cell Development. Annu. Rev. Immunol. *34*, 93–119.

Naik, S.H., Proietto, A.I., Wilson, N.S., Dakic, A., Schnorrer, P., Fuchsberger, M., Lahoud, M.H., O'Keeffe, M., Shao, Q.X., Chen, W.F., et al. (2005). Cutting edge: generation of splenic CD8+ and CD8- dendritic cell equivalents in Fms-like tyrosine kinase 3 ligand bone marrow cultures. J. Immunol. *174*, 6592–6597.

Ouyang, X., Zhang, R., Yang, J., Li, Q., Qin, L., Zhu, C., Liu, J., Ning, H., Shin, M.S., Gupta, M., et al. (2011). Transcription factor IRF8 directs a silencing programme for TH17 cell differentiation. Nat. Commun. *2*, 314.

Palucka, K., and Banchereau, J. (2013). Dendritic-cell-based therapeutic cancer vaccines. Immunity *39*, 38–48.

Poulin, L.F., Salio, M., Griessinger, E., Anjos-Afonso, F., Craciun, L., Chen, J.L., Keller, A.M., Joffre, O., Zelenay, S., Nye, E., et al. (2010). Characterization of human DNGR-1+ BDCA3+ leukocytes as putative equivalents of mouse CD8alpha+ dendritic cells. J. Exp. Med. *207*, 1261–1271.

Proietto, A.I., Mittag, D., Roberts, A.W., Sprigg, N., and Wu, L. (2012). The equivalents of human blood and spleen dendritic cell subtypes can be generated in vitro from human CD34(+) stem cells in the presence of fms-like tyrosine kinase 3 ligand and thrombopoietin. Cell. Mol. Immunol. *9*, 446–454.

Pulendran, B. (2015). The varieties of immunological experience: of pathogens, stress, and dendritic cells. Annu. Rev. Immunol. *33*, 563–606.

Quinlan, A.R., and Hall, I.M. (2010). BEDTools: a flexible suite of utilities for comparing genomic features. Bioinformatics *26*, 841–842.

Radtke, F., MacDonald, H.R., and Tacchini-Cottier, F. (2013). Regulation of innate and adaptive immunity by Notch. Nat. Rev. Immunol. *13*, 427–437.

Redecke, V., Wu, R., Zhou, J., Finkelstein, D., Chaturvedi, V., High, A.A., and Häcker, H. (2013). Hematopoietic progenitor cell lines with myeloid and lymphoid potential. Nat. Methods *10*, 795–803.

Roberts, E.W., Broz, M.L., Binnewies, M., Headley, M.B., Nelson, A.E., Wolf, D.M., Kaisho, T., Bogunovic, D., Bhardwaj, N., and Krummel, M.F. (2016). Critical Role for CD103(+)/CD141(+) Dendritic Cells Bearing CCR7 for Tumor Antigen Trafficking and Priming of T Cell Immunity in Melanoma. Cancer Cell *30*, 324–336.

Salmon, H., Idoyaga, J., Rahman, A., Leboeuf, M., Remark, R., Jordan, S., Casanova-Acebes, M., Khudoynazarova, M., Agudo, J., Tung, N., et al. (2016). Expansion and Activation of CD103(+) Dendritic Cell Progenitors at the Tumor Site Enhances Tumor Responses to Therapeutic PD-L1 and BRAF Inhibition. Immunity *44*, 924–938.

Satpathy, A.T., Briseño, C.G., Lee, J.S., Ng, D., Manieri, N.A., Kc, W., Wu, X., Thomas, S.R., Lee, W.L., Turkoz, M., et al. (2013). Notch2-dependent classical dendritic cells orchestrate intestinal immunity to attaching-and-effacing bacterial pathogens. Nat. Immunol. *14*, 937–948.

Schmitt, T.M., de Pooter, R.F., Gronski, M.A., Cho, S.K., Ohashi, P.S., and Zúñiga-Pflücker, J.C. (2004). Induction of T cell development and establishment of T cell competence from embryonic stem cells differentiated in vitro. Nat. Immunol. *5*, 410–417.

Schraml, B.U., and Reis e Sousa, C. (2015). Defining dendritic cells. Curr. Opin. Immunol. *32*, 13–20.

Sichien, D., Scott, C.L., Martens, L., Vanderkerken, M., Van Gassen, S., Plantinga, M., Joeris, T., De Prijck, S., Vanhoutte, L., Vanheerswynghels, M., et al. (2016). IRF8 Transcription Factor Controls Survival and Function of Terminally Differentiated Conventional and Plasmacytoid Dendritic Cells, Respectively. Immunity *45*, 626–640.

Spranger, S., Dai, D., Horton, B., and Gajewski, T.F. (2017). Tumor-Residing Batf3 Dendritic Cells Are Required for Effector T Cell Trafficking and Adoptive T Cell Therapy. Cancer Cell *31*, 711–723.e4.

Steinman, R.M. (2012). Decisions about dendritic cells: past, present, and future. Annu. Rev. Immunol. *30*, 1–22.

Worbs, T., Hammerschmidt, S.I., and Förster, R. (2017). Dendritic cell migration in health and disease. Nat. Rev. Immunol. *17*, 30–48.

Wu, Y., Cain-Hom, C., Choy, L., Hagenbeek, T.J., de Leon, G.P., Chen, Y., Finkle, D., Venook, R., Wu, X., Ridgway, J., et al. (2010). Therapeutic antibody targeting of individual Notch receptors. Nature *464*, 1052–1057.

Yamashiro, S. (2012). Functions of fascin in dendritic cells. Crit. Rev. Immunol. *32*, 11–21.

Zhan, Y., Carrington, E.M., van Nieuwenhuijze, A., Bedoui, S., Seah, S., Xu, Y., Wang, N., Mintern, J.D., Villadangos, J.A., Wicks, I.P., and Lew, A.M. (2011). GM-CSF increases cross-presentation and CD103 expression by mouse $CD8^+$ spleen dendritic cells. Eur. J. Immunol. *41*, 2585–2595.

STAR★METHODS

KEY RESOURCES TABLE

REAGENT or RESOURCE	SOURCE	IDENTIFIER
Antibodies		
Rat anti-mouse CD45 (clone 30-F11)	BD Biosciences	Cat# 550994
Rat anti-mouse CD205 (clone NLDC-145)	BioLegend	Cat# 138207
Rat anti-mouse CD317 (Bst2) (clone 927)	BioLegend	Cat# 127012
Rat anti-mouse CD205 (clone NLDC-145)	BioLegend	Cat# 138209, 138205
Rat anti-mouse/human CD45R/B220 (clone RA3-6B2)	BioLegend	Cat# 103232
Rat anti-mouse CD127 (clone A7R34)	eBioscience	Cat# 11-1271-81
Armenian hamster anti-mouse CD11c (clone N418)	eBioscience	Cat# 11-0114-82, 13-0114-85
Rat anti-mouse CD4 (clone GK1.5)	eBioscience	Cat# 11-0041-82
Rat anti-mouse CD135 (FLT3) (clone A2F10)	eBioscience	Cat# 46-1351-80
Rat anti-mouse CD8a (clone 53-6.7)	eBioscience	Cat# 45-0081-82, 17-0081-83
Rat anti-mouse/human CD44 (clone IM7)	eBioscience	Cat# 17-0441-83
Rat anti-mouse CD25 (clone PC61.5)	eBioscience	Cat# 12-0251-82
Rat anti-mouse CD11b (clone M1/70)	eBioscience	Cat# 47-0112-82, 25-0112-81
Rat anti-mouse CD117 (c-Kit) (clone 2B8)	eBioscience	Cat# 47-1171-80
Armenian hamster anti-mouse TCR beta (clone H57-597)	eBioscience	Cat# 47-5961-82
Syrian hamster anti-mouse CD3e (clone eBio500A2)	eBioscience	Cat# 48-0033-82
Rat anti-mouse CD24 (clone M1/69)	eBioscience	Cat# 12-0242-81
Rat anti-mouse ESAM (clone 1G8)	eBioscience	Cat# 12-5852-81
Rat anti-mouse CD115 (c-fms) (clone AFS98)	eBioscience	Cat# 12-1152-81
Armenian hamster anti-mouse CD103 (clone 2E7)	eBioscience	Cat# 12-1031-81
Rat anti-mouse/human CD45R (B220) (clone RA3-6B2)	eBioscience	Cat# 25-0452-82
Rat anti-mouse CD4 (clone RM4-5)	eBioscience	Cat# 25-0042-81
Rat anti-mouse MHC Class II (I-A/I-E) (clone M5/114.15.2)	eBioscience	Cat# 56-5321-82
Mouse anti-mouse NK1.1 (clone PK136)	eBioscience	Cat# 48-5941-80
Rat anti-mouse TER-119 (clone TER-119)	eBioscience	Cat# 48-5921-82
Rat anti-mouse Ly-6G (Gr-1) (clone RB6-8C5)	eBioscience	Cat# 48-5931-82
Rat anti-mouse CCR7 (clone 4B12)	eBioscience	Cat# 12-1971-82
Anti-mouse Clec9a (clone 42D2)	eBioscience	Cat# 12-5975-80
Anti-mouse Xcr1 (clone ZET)	BioLegend	Cat# 148207
Anti-mouse CD172a (clone P84)	BD Biosciences	Cat# 560107
Synthetic human anti-mouse/human NOTCH2 negative regulatory region (NRR)	(Wu et al., 2010)	N/A
Mouse anti-human CD11c (AF700/BV605/BV711 conjugate, clone B-ly6, 3/5/5 µl/50 µl sample)	BD/BD/Biolegend	Cat# 561352, 563929, 301630
Mouse anti-human CD123 (BV421/BUV395 conjugate, clone 6H6/7G3, 3/5 µl/50 µl sample)	Biolegend/BD	Cat# 306018, 564195
Mouse anti-human CD14 (BV650 conjugate, clone M5E2, 4 µl/50 µl sample)	Biolegend	Cat# 301835
Mouse anti-human CD141 (BV510/APC conjugate, clone 1A4/AD5-14H12, 3/5 µl/50 µl sample)	BD/Miltenyi	Cat# 563298, 130-090-907
Mouse anti-human CD15 (BV605 conjugate, clone W6D3, 3 µl/50 µl sample)	BD	Cat# 562980
Mouse anti-human CD16 (FITC/AF700 conjugate, clone 3G8, 3/1 µl/50 µl sample)	BD/Biolegend	Cat# 335035, 302026

(*Continued on next page*)

Continued		
REAGENT or RESOURCE	SOURCE	IDENTIFIER
Mouse anti-human CD19 (FITC/AF700 conjugate, clone 4G7/HIB19, 3/1 μl/50 μl sample)	BD/Biolegend	Cat# 345776, 302226
Mouse anti-human CD1c (PE-Cy7/PerCP-Cy5.5 conjugate, clone L161, 3/5 μl/50 μl sample)	Biolegend	Cat# 331516, 331513
Mouse anti-human CD20 (FITC/AF700 conjugate, clone L27/2H7, 3/1 μl/50 μl sample)	BD/Biolegend	Cat# 345792, 302322
Mouse anti-human CD3 (FITC/AF700 conjugate, clone SK7 (Leu-4), 3/5 μl/50 μl sample)	BD/Biolegend	Cat# 345763, 344822
Mouse anti-human CD303 (BDCA-2) (APC/BV605 conjugate, clone 201A, 3/5 μl/50 μl sample)	Biolegend	Cat# 354206, 354224
Mouse anti-human CD304 (APC/BV605 conjugate, clone 12C2/U21-1283, 3/5 μl/50 μl sample)	Biolegend/BD	Cat# 354506, 743130
Mouse anti-human CD34 (FITC conjugate, clone 8G12, 3 μl/50 μl sample)	BD	Cat# 345801
Mouse anti-human CD4 (BV421 conjugate, clone RPA-T4, 5 μl/50 μl sample)	Biolegend	Cat# 300531
Mouse anti-human CD45 (APC-Cy7 conjugate, clone 2D1, 3 μl/50 μl sample)	BD	Cat# 557833
Mouse anti-human CD56 (FITC conjugate, clone NCAM16.2, 3 μl/50 μl sample)	BD	Cat# 345811
Mouse anti-human CD8 (APC-Cy7 conjugate, clone SK1, 5 μl/50 μl sample)	BD	Cat# 557834
Mouse anti-human CLEC9A (CD370, DNGR1) (PE conjugate, clone 8F9, 3 μl/50 μl sample)	Biolegend	Cat# 353804
Mouse anti-human HLA-DR (PerCP-Cy5.5/BV785/V500 conjugate, clone L243/L243/G46-6, 3/5/5 μl/50 μl sample)	BD/Biolegend/BD	Cat# 339216, 307642, 561224
Mouse anti-human IL-12p40/p70 (BV421 conjugate, clone C8.6, 5 μl/50 μl sample)	BD	Cat# 565023
Mouse anti-human IFN-a (PE conjugate, clone LT27:295, 10 μl/50 μl sample)	Milteyni	Cat# 130-092-601
Mouse anti-human TNF-a (APC-Cy7 conjugate, clone Mab11, 5 μl/50 μl sample)	Biolegend	Cat# 502944
Chemicals, Peptides, and Recombinant Proteins		
DAPI	Sigma-Aldrich	Cat# D8417
Permeabilization buffer	eBioscience	Cat# 00-8333-56
Paraformaldehyde (PFA) 4% in PBS	Affymetrix	Cat# 19943 1LT
Mitomycin C from *Streptomyces caespitosus*	Sigma-Aldrich	Cat# T5648
CFSE	Invitrogen	Cat# C34554
Brefeldin A	Sigma-Aldrich	Cat# B7651
Streptavidin PerCP-Cyanine5.5	eBioscience	Cat# 45-4317-82
Streptavidin APC	eBioscience	Cat# 17-4317-82
Fixable Viability Dye eFluor 506	eBioscience	Cat# 65-0866-14
iTAg Tetramer/PE - H-2 Kb OVA (SIINFEKL)	MBL International	Cat# TB-5001-1
Collagenase D	Sigma-Aldrich	Cat# COLLD-RO
ChromPure mouse IgG	Jackson Laboratories	Car# 015-000-003
Deoxyribonuclease I (DNase I) from bovine pancreas	Sigma-Aldrich	Cat# D5025
Ovalbumin from chicken egg white, endotoxin-free (OVA)	InvivoGen	Cat# vac-ova
Streptavidin microbeads	Miltenyi Biotec	Cat# 130-048-101
DNase I	QIAGEN	Cat# 79254
Recombinant murine SLC (CCL21)	Peprotech	Cat# 250-13
Recombinant murine RANTES (CCL5)	Peprotech	Cat# 250-07

(*Continued on next page*)

Continued

REAGENT or RESOURCE	SOURCE	IDENTIFIER
Recombinant murine MCP-1 (CCL2)	Peprotech	Cat# 250-10
Recombinant murine MIP-3β (CCL19)	Peprotech	Cat# 250-27B
FCS, charcoal stripped	GIBCO	Cat# 12676-029
TRIzol LS Reagent	Invitrogen	Cat# 10296028
Critical Commercial Assays		
ARCTURUS PicoPure RNA Isolation Kit	Applied Biosystems	Cat# KIT0204
Deposited Data		
RNA-seq analysis of cultured dendritic cells	This paper	GEO: GSE110577
RNA-seq analysis of primary splenic dendritic cells	(Lau et al., 2016)	GEO: GSE76132
Microarray analysis of *Notch2*-deficient dendritic cells	(Satpathy et al., 2013)	GEO: GSE45681
Experimental Models: Cell Lines		
Mouse: OP9-GFP	(Mohtashami et al., 2016)	N/A
Mouse: OP9-DL1	(Mohtashami et al., 2016)	N/A
Mouse: OP9-DL4	(Mohtashami et al., 2016)	N/A
Mouse: B16-OVA	(Falo et al., 1995)	N/A
Mouse: B16-FLT3L	(Mach et al., 2000)	N/A
Mouse: HoxB8-FL	(Redecke et al., 2013)	N/A
Experimental Models: Organisms/Strains		
Mouse: C57BL/6	The Jackson Laboratory	Stock #000664
Mouse: Rag2/OT-I	Taconic	2334
Mouse: $Irf8^{-/-}$:B6(Cg)-$Irf8^{tm1.2Hm}$/J	(Ouyang et al., 2011)	N/A
Mouse: $Irf8^{flox}$: B6(Cg)-$Irf8^{tm1.1Hm}$/J	(Feng et al., 2011)	N/A
Mouse: *Itgax-Cre*: B6.Cg-Tg(Itgax-cre)1-1Reiz/J	(Caton et al., 2007)	N/A
Mouse: $Batf3^{-/-}$: B6.129S(C)-$Batf3^{tm1Kmm}$/J	(Hildner et al., 2008)	N/A
Software and Algorithms		
FlowJo v9.9.5 and v10.1	FlowJo, LLC	https://www.flowjo.com/
Prism 7	GraphPad	https://www.graphpad.com/
DESeq2 (v3.0)	(Love et al., 2014)	http://bioconductor.org/packages/release/bioc/html/DESeq2.html
R (v.3.3.2).	N/A	https://www.r-project.org/
STAR anligner (v2.5.0c)	(Dobin et al., 2013)	https://github.com/alexdobin/STAR
Picard tools (v.1.126)		http://broadinstitute.github.io/picard/
HTSeq (v0.6.0)	(Anders et al., 2015)	https://github.com/simon-anders/htseq
BEDTools (v2.17.0)	(Quinlan and Hall, 2010)	https://github.com/arq5x/bedtools2
nSolver (advanced analysis module v.1.1.4)	Nanostring	https://www.nanostring.com/products/analysis-software/nsolver

CONTACT FOR REAGENT AND RESOURCE SHARING

Further information and requests for resources and reagents should be directed to and will be fulfilled by the Lead Contact, Boris Reizis (Boris.Reizis@nyumc.org).

EXPERIMENTAL MODEL AND SUBJECT DETAILS

Human studies

The study was performed in accordance with the Declaration of Helsinki. Written informed consent was obtained from participants prior to recruitment. The study was approved by NRES Committee North East-Newcastle and North Tyneside (08/H0906/72, 14/NE/1212 and 14/NE/1136). Peripheral blood or bone marrow was obtained from healthy volunteers or from the previously described patients with biallelic *IRF8* mutations (Bigley et al., 2017; Hambleton et al., 2011).

Animals

All animal studies were performed according to the investigator's protocol approved by the Institutional Animal Care and Use Committees of New York University School of Medicine and of Columbia University Medical Center. Wild-type C57BL/6 mice (Jackson Laboratories) and Rag2-deficient OT-I TCR transgenic mice (Rag2/OT-I, Taconic) were maintained by intercrossing in the animal facility at New York University School of Medicine. Mice deficient for *Batf3* (Hildner et al., 2008) or Irf8 (Ouyang et al., 2011), mice with a conditional LoxP-flanked allele of *Irf8* (*Irf8*^{flox}) (Feng et al., 2011) and the *Itgax*-Cre deleter strain (Caton et al., 2007) have been described previously and were on pure C57BL/6 background. *Irf8*^{flox} and *Itgax*-Cre mice were intercrossed to obtain *Irf8*$^{flox/flox}$ *Itgax*-Cre^{+} mice with a specific deletion of Irf8 in CD11c^{+} cells. Mice were group-housed in individually ventilated cages and maintained under specific pathogen-free conditions. Male and female mice were used between 8 and 16 weeks of age. No obvious difference between sexes was observed within the parameters analyzed for our experiments.

Cell lines

FLT3L-secreting (Mach et al., 2000) and OVA-expressing (Falo et al., 1995) clones of the C57BL/6-derived B16 melanoma cell line (B16-FLT3L and B16-OVA, respectively) were cultured in DMEM medium supplemented with 10% fetal calf serum (FCS), 1% L-glutamine, 1% sodium pyruvate, 1% MEM-NEAA and 1% penicillin/streptomycin (full DMEM) at 37°C in a humidified atmosphere at 5% CO_2. The murine progenitor Hoxb8-FL cell line (Redecke et al., 2013) was cultured in RPMI medium supplemented with 10% FCS, 1% L-glutamine, 1% penicillin/streptomycin, 10% supernatant from cultured B16-FLT3L cell line and 1 μm β-estradiol at 37°C in a humidified atmosphere at 5% CO_2. For differentiation, Hoxb8-FL cells were cultured in the same medium without estradiol and with charcoal-stripped FCS to ensure the absence of estradiol. OP9 cell lines transduced with retroviruses encoding green fluorescent protein (GFP) or Notch ligands DL1 or DL4 (Mohtashami et al., 2016; Schmitt et al., 2004) were cultured in MEM-α medium supplemented with 20% FCS and 1% penicillin/streptomycin (OP9 medium) at 37°C in a humidified atmosphere at 5% CO_2. Prior to use in co-cultures, OP9 cells were treated with mitomycin C at 10 μg/mL for 2 hr, harvested, washed three times in PBS and resuspended in OP9 medium. For co-culture with human cells, OP9 were maintained in MEM-α supplemented with 10% FCS and 1% penicillin/streptomycin.

METHOD DETAILS

Methods: mouse

Cell preparations

Spleens were minced and digested with collagenase D (1 mg/mL) and DNase I (20 μg/mL) in full DMEM for 30 min at 37°C. Tissues were pressed through a nylon 70 μm cell strainer to yield single-cell suspensions and then subjected to red blood cell (RBC) lysis (155 mM NH_4Cl, 10 mM $NaHCO_3$, 0.1 mM EDTA) for 5 min at room temperature before being filtered. Bone marrow (BM) was prepared by flushing femurs and tibias with phosphate buffer saline (PBS) using a 27-gauge needle followed by RBC lysis and filtering through a sterile 70 μm cell strainer. Peripheral blood (PB) was obtained by submandibular bleed and subjected to RBC lysis for 5 minutes, followed by 3 min, at room temperature (RT).

Flow cytometry

Single-cell suspensions of cultured DCs or primary cells were stained for multicolor analysis with the indicated fluorochrome- or biotin-conjugated antibodies. Antibodies were diluted in FACS buffer (PBS, 1% FCS, 0.02% NaN_3). With the exception of Hoxb8-derived DCs, staining of surface molecules with fluorescently labeled antibodies was performed for 20 min at 4°C in the dark. Hoxb8-derived DCs were stained at room temperature. For *in vivo* cross-presentation experiments, the OVA peptide/H-2K^{b} tetramer was used and staining was performed for 30 min at room temperature. Samples were acquired on LSR II (BD) flow cytometer using FACSDiva software (BD Biosciences) or Attune NxT (Invitrogen) using Attune NxT software and further analyzed with FlowJo software (Tree Star).

FLT3L-driven DC differentiation of Hoxb8-FL cultures

Hoxb8-FL progenitor cells were expanded in culture and differentiated as previously described (Grajkowska et al., 2017; Redecke et al., 2013). Briefly, progenitor cells were removed from β-estradiol-supplemented medium and washed three times in PBS with 10% charcoal-stripped FBS at room temperature. The cells were then plated in fresh medium without estradiol at $2x10^5$ cells per well in 6-well plates and cultured for 7 days without replating.

Notch-driven DC differentiation of Hox8-FL cultures

FLT3L-driven Hoxb8-FL DC differentiation was initiated as described above. On day 3 of differentiation, cells were harvested and resuspended in fresh medium. The cells were then plated at $2.5x10^5$ cells per well in 24-well plates containing a monolayer of mitomycin-treated OP9-DL1 cells. Where indicated, control OP9-GFP or OP9-DL4 cells were used in a similar fashion. For NOTCH2 blocking experiments, cells were treated with anti-NOTCH2 antibody or control IgG (50 ng/mL or 500 ng/mL) on day 3 at the time of co-culture initiation. Cell cultures were analyzed by flow cytometry on day 7.

FLT3L-driven DC differentiation of primary BM cultures

Single cell suspensions of primary murine BM cells were obtained as described above. The cells were suspended in DMEM medium supplemented with 10% FCS, 1% L-glutamine, 1% sodium pyruvate, 1% MEM-NEAA and 1% penicillin/streptomycin, 55 μM 2-mercaptoethanol and, 10% supernatant from cultured B16-FLT3L cell line (DC medium). Cells were plated at $2x10^6$ cells

per well in 2 mL of DC medium in 24-well plates and cultured at 37°C in a humidified atmosphere at 5% CO_2 for 7 days without replating.

Notch-driven DC differentiation of primary BM cultures

Primary BM cultures were initiated as described above. On day 3 of differentiation, half of the volume of cells in DC medium from each well was transferred to a single well containing a monolayer of mitomycin-treated OP9 cells in 24-well plates. Where indicated, control OP9-GFP or OP9-DL4 cells were used in a similar fashion. For NOTCH2 blocking experiments, cells were treated with anti-NOTCH2 antibody or control IgG at various concentrations on day 3 at the time of coculture initiation. Cell cultures were analyzed on day 7.

In vitro DC migration assay

DC migratory capacity was evaluated using a transwell assay using 24-well plates of 6.5 mm transwells with 5.0 μm pore polycarbonate membrane (Corning). Cultured DCs were harvested on day 7 and resuspended in DMEM medium supplemented with 2% FCS, 1% L-glutamine, 1% sodium pyruvate, 1% MEM-NEAA and 1% penicillin/streptomycin (migration medium) at 5×10^5 cells/ml. Migration medium containing chemokines (100 ng/mL in 0.6 mL total volume) was placed at the bottom of each well. DC suspension (100 μL) was added to the top chamber and incubated at 37°C in a humidified atmosphere at 5% CO_2 for 3 hr. Migrated cells at the bottom of the wells were recovered in 500 μL of cold PBS supplemented with 2% FBS and 0.5 mM EDTA and analyzed by flow cytometry. For each DC type, frequency of migrated cells was calculated as # cells migrated out of total input per DC type as determined flow cytometry.

In vitro T cell stimulation

Endotoxin-free chicken ovalbumin (OVA) protein (Sigma Aldrich) was added to DC cultures on day 7 to a final concentration of 100 μg/ml. Cells were then harvested, washed three times with PBS and filtered using sterile 70 μm cell strainers. Single cells suspensions were obtained from the spleens and lymph nodes of Rag2/OT-I mice as described above. $CD8^+$ T cells were isolated by magnetic-activated cell separation (MACS) by negative selection using biotinylated antibodies (B220, Ter119, Gr1, CD11c, NK1.1, F4/80, CD4, DX5) with streptavidin microbeads, and MACS columns (Miltenyi Biotec). $CD8^+$ T cells were stained with carboxyfluorescein succinimidyl ester (CFSE, 5 μM), washed and $4x10^4$ T cells were cultured with DCs at 1:1-10:1 ratio for 3 days. T cell proliferation was assessed by CFSE dilution using flow cytometry.

In vivo cross presentation assay

DC cultures were pulsed with endotoxin-free OVA protein for 2 hr as above. Cells were pooled, harvested, washed three times with PBS and filtered twice using sterile 70 μm cell strainers. To enrich cDC1, cells were stained with biotinylated antibodies against B220 and CD172a (FL cultures) or B220 and CD11b (FL+Notch cultures), and purified by negative selection on MACS columns. OVA-pulsed total DCs ($2.5x10^5$ - $1x10^6$) or OVA-pulsed enriched cDC1 ($3x10^5$) were resuspended in 0.1 mL PBS and injected i.v. into the retroorbital sinus. Spleens were harvested or PB was collected and analyzed by flow cytometry using OVA peptide/H-$2K^b$ tetramer staining 7 days after DC vaccination.

Tumor challenge

B16-OVA melanoma cells were harvested, washed twice with PBS, and filtered. A total of $2.5x10^5$ B16-OVA cells in 0.1 mL PBS were injected i.v. into the retroorbital sinus 7-10 days following vaccination with OVA-pulsed DCs. Mice were monitored daily and sacrificed when moribund or on day 25 of observation if healthy. Lungs from perfused mice were harvested, fixed in 4% paraformaldehyde for 24 hr, sectioned and stained with hematoxylin/eosin for histological examination using light microscopy. Where indicated, $4x10^5$ B16-OVA cells in 0.2 mL PBS were injected i.v. into the tail vein.

Cell sorting and sample preparation for RNA-seq

DC populations were stained and sorted on BD FACSAria II as follows: cDC1 ($CD11c^{hi}$ $MHCII^+$ $B220^-$ $CD24^+$) and cDC2 ($CD11c^{hi}$ $MHCII^+$ $B220^-$ $CD11b^+$). Sorted cells ($1–3x10^5$) were resuspended in 750 μL Trizol LS (Invitrogen), and RNA was extracted using the Arcturus PicoPure kit (Thermo Fisher Scientific). Equal volume of 70% ethanol was added to the aqueous phase of TRIzol samples and applied to columns from the PicoPure kit. Up to 250 μL of ethanol/aqueous phase mix was loaded onto the column and spun at 100 *g* for 2 min for each load. Bound RNA was washed, treated with DNase I (QIAGEN), and eluted as per manufacturer's instructions. To remove phenol contamination, eluate was resuspended in 100 μL of Wash Buffer 1 and reloaded onto a fresh column followed by elution. RNASeq libraries were prepared using the Clontech Ultra low RNA kit, starting with 3 ng, with 10 cycles of PCR for cDNA amplification, and the Clontech Low Input Kit for library prep, with 7 cycles of PCR amplification, following the manufacturer's protocol. The amplified library was purified using AMPure beads, quantified by Qubit and QPCR, and visualized in an Agilent Bioanalyzer. The libraries were pooled equimolarly, and run on a HiSeq 2500 as paired, 50 nucleotide in length.

RNA-Seq data processing

Sequencing reads were mapped to the mouse reference genome (GRCm38.85/mm10) using the STAR aligner (v2.5.0c) (Dobin et al., 2013). Alignments were guided by a Gene Transfer Format (Ensembl GTF GRCm38.85). The mean read insert sizes and their standard deviations were calculated using Picard tools (v.1.126) (http://broadinstitute.github.io/picard/). The read count tables were generated using HTSeq (v0.6.0) (Anders et al., 2015), normalized based on their library size factors using DESeq2 (v3.0) (Love et al., 2014), and differential expression analysis was performed. The Read Per Million (RPM) normalized BigWig files were generated using BEDTools (v2.17.0) (Quinlan and Hall, 2010) and bedGraphToBigWig tool (v4), and downstream statistical analyses and generating plots were performed in R environment (v3.1.1) (http://www.r-project.org/).

Methods: human

Cell isolation, flow cytometry and cell sorting

Peripheral blood or bone marrow mononuclear cells (PBMC) were isolated by density centrifugation. For flow cytometry or fluorescence-activated cell sorting (FACS) (purity > 98%), cells were stained in aliquots of 1-3 x10^6 cells/50 μl of Dulbecco's phosphate-buffered saline with 0.1%–2% fetal calf serum and 0.4% EDTA. Dead cells, usually < 5%, were excluded by DAPI (Partec) or Zombie (Biolegend) staining. Analysis was performed with an LSRFortessa X-20 and sorting with a FACSAria III (BD Biosciences) running BD FACSDIVA 8.0.1 or 8.0 software, respectively. Data were processed with FlowJo 10.4.1 (Treestar, Inc). Intracellular staining was performed after surface staining, lysis, and fixation (eBioscience) according to manufacturer's instructions.

In vitro *generation of dendritic cells*

FACS-purified $CD34^+$ bone marrow progenitors were cultured (typically 3000/well) in 96 well U-bottomed plates with or without pre-seeded OP9, OP9-DL1 or OP9-DL4 stromal cells (5000/well). Culture media consisted of 200 μL α-MEM (GIBCO) supplemented with 1% penicillin/streptomycin (Sigma), 10% fetal calf serum (GIBCO), 20 ng/ml granulocyte-macrophage colony-stimulating factor (GM-CSF, R&D systems), 100 ng/ml FLT3-ligand (FL, Immunotools), 20 ng/ml stem cell factor (SCF, Immunotools). Half the volume of media, with cytokines, was replaced weekly. At day 14 or 21, cells were harvested on ice, passed through a 50 μm filter, washed and stained for flow cytometric analysis or cell sorting. Cell output was normalized to 3000 input progenitors per well.

Dendritic cell functional analysis

For cytokine production, PBMC from healthy controls or *in vitro*-generated cells were cultured in the presence of polyinosinic: polycytidylic acid (poly(I:C), 10 μg/ml, Invivogen), Lipopolysaccharide (LPS, 5ng/ml, Sigma), CL075 (1 μg/ml, Invivogen) and CpG (ODN 2216, 7.5 μM, Invivogen) for 14h at 37°C, 5% CO_2 with addition of Brefeldin A (10 μg/ml, eBioscience) after 3 hr. Dead cells (usually < 30%) were excluded with Zombie amine dye (Biolegend). Intracellular cytokine staining was performed after surface staining, fixation, and permeabilization (eBIoscience) according to manufacturer's instructions.

For T cell proliferation, FACS-purified *ex vivo* or *in vitro* generated DCs (2,500-8,000 DC/well) were cultured with FACS purified allogeneic $CD3^+$ T cells at a ratio of 1:10 DC:T cell (n = 2-9 DC/T cell pairs). Positive controls were generated by T cell co-culture with CD3+CD28+ beads (Dynabeads®, Thermo Fisher Scientific) at T cell:bead ratio 1:1. T cell proliferation was assessed by CFSE dilution on day 5 of culture.

NanoString nCounter Gene expression analysis

Ex vivo or culture-generated DCs were FACS purified (> 98% purity) and lysed in RLT buffer containing 1% β-mercaptoethanol, at a concentration of 2000 cells/μl. Samples were analyzed on the NanoString nCounter® FLEX platform according to manufacturer's instructions. Briefly, 5 μl of lysate (10,000 cells) was mixed with reporter probes, hybridization buffer, and capture probes and hybridized at 65°C for 12-30 hr. Samples were then processed on the NanoString Prep station and cartridges were read on the NanoString Digital Analyzer to yield a reporter code count (RCC) dataset. The human Immunology_V2 panel was used, supplemented with the following 30 genes: *ASIP, DAXX, MERTK, C19orf59, DBN1, Ki67, CCL17, F13A1, NDRG2, CD1c, FGD6, PACSIN1, CD207, FLT3, PPM1N, CLEC10A, GCSAM, PRAM1, CLEC9A, GGT5, S100A12, CLNK, LPAR2, TMEM14A, COBLL1, LYVE1, UPK3A, CXCL5, MAFF, ZBTB46.*

Counts were normalized within the nSolver software (advanced analysis module version 1.1.4). The log2 transformed output data were analyzed using R (version 3.3.3). For principal component analysis (PCA), genes expressed below $2^{4.8}$ in all samples were removed (167/608). A culture signature was derived by performing pairwise comparisons (two-tailed t test with Benjamini-Hochberg correction of p values) of all culture versus all *ex vivo* populations. 102 genes with adjusted p values < 0.05 (the culture signature) were excluded from further analysis. The remaining 339 genes were used to construct the PCA plot. Heatmaps were generated in R and display log2 transformed expression.

QUANTIFICATION AND STATISTICAL ANALYSIS

For experimental results, all statistical calculations analysis were performed using Prism (GraphPad, La Jolla, CA). In animal experiments, statistical significance of differences between experimental groups was determined by non-parametric Mann-Whitney test. Differences in the Kaplan-Meier survival plots (Figure 5) were analyzed using log-rank test. In human cell culture experiments, statistical significance of differences between experimental groups was determined by unpaired, two-tailed Student's t test.

For RNA-seq data, Wald test p values and adjusted p values provided by DESeq2 package were used for differential expression analysis (adjusted $p < 0.1$, FC > 2) which is based on estimating dispersions and uses a negative binomial generalized linear model. For sample clustering, we performed a classical multidimensional scaling (MDS) and a Euclidean distance based clustering. For pathway and enrichment analysis, we used hypergeometric distribution tests performed by clusterProfiler package (adjusted $p < 0.1$). The datasets were individually and comprehensively analyzed and visualized all in the R statistical environment (v3.2.5).

DATA AND SOFTWARE AVAILABILITY

The complete processed expression data from RNA-seq and Nanostring experiments are attached as Tables S1 and S5, respectively. The accession number for the raw RNA-seq sequencing data reported in this paper is GEO: GSE110577.

Cell Reports, Volume *23*

Supplemental Information

Notch Signaling Facilitates *In Vitro* Generation of Cross-Presenting Classical Dendritic Cells

Margaret E. Kirkling, Urszula Cytlak, Colleen M. Lau, Kanako L. Lewis, Anastasia Resteu, Alireza Khodadadi-Jamayran, Christian W. Siebel, Hélène Salmon, Miriam Merad, Aristotelis Tsirigos, Matthew Collin, Venetia Bigley, and Boris Reizis

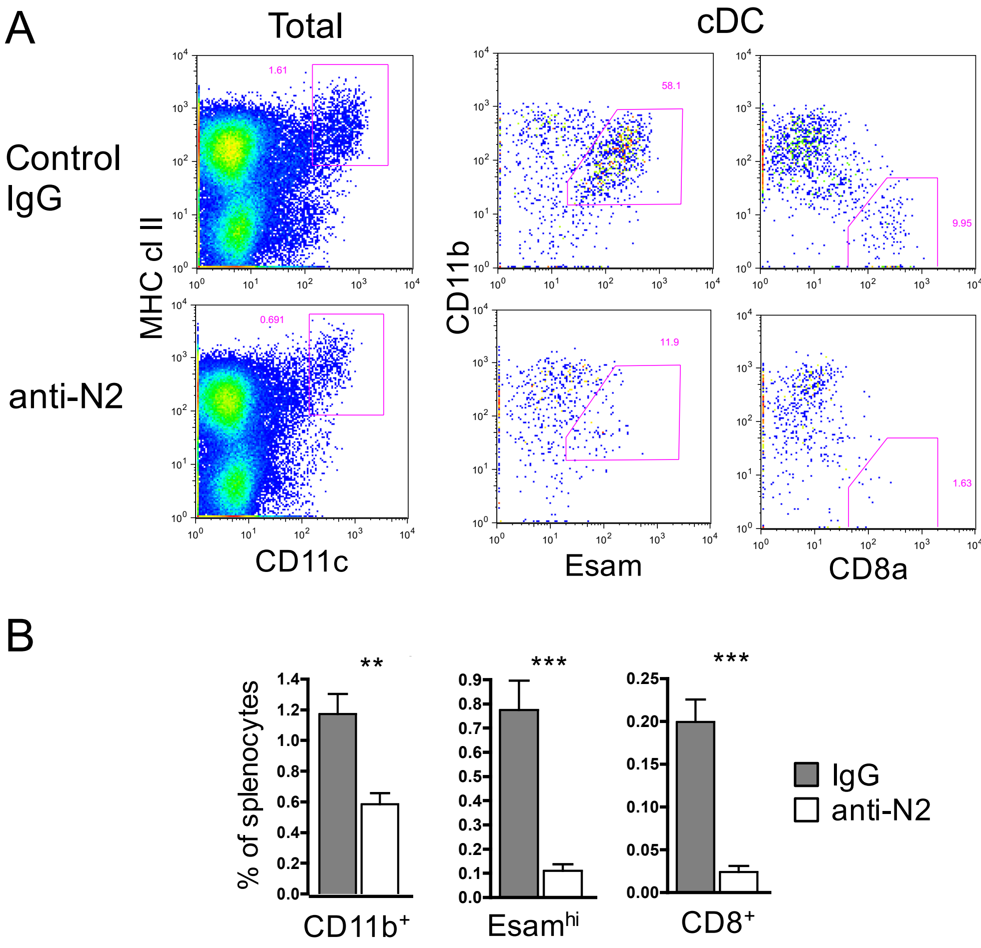

Figure S1. The effect of antibody-mediated Notch2 blockade in vivo. Related to Figure 1.
Wild-type animals were administered anti-N2 or control IgG 5 times every 4 days, and analyzed 4 days after the last administration.
A. Representative staining profiles of splenic DC. Shown are total splenocytes with CD11c^{hi} MHC cl II^{+} cDC highlighted; and gated cDC with CD11b^{+} Esam^{hi} cDC2 and CD11b^{-} CD8α^{+} cDC1 highlighted. Note the appearance of CD11b^{-} CD8α$^{-/lo}$ DC that were also previously observed upon genetic Notch2 deletion (Lewis et al., 2011; Satpathy et al., 2013) and likely represent cDC1 that lost CD8α expression.
B. The fraction of total CD11b^{+} cDC2, CD11b^{+} Esam^{hi} cDC2 and CD8α^{+} cDC1 among total splenocytes. Data represent mean ± S.D. of 3 animals from one experiment; representative of 3 experiments.
Statistical significance was estimated using Student's *t*-test: ***, $p<0.001$; **, $p<0.01$.

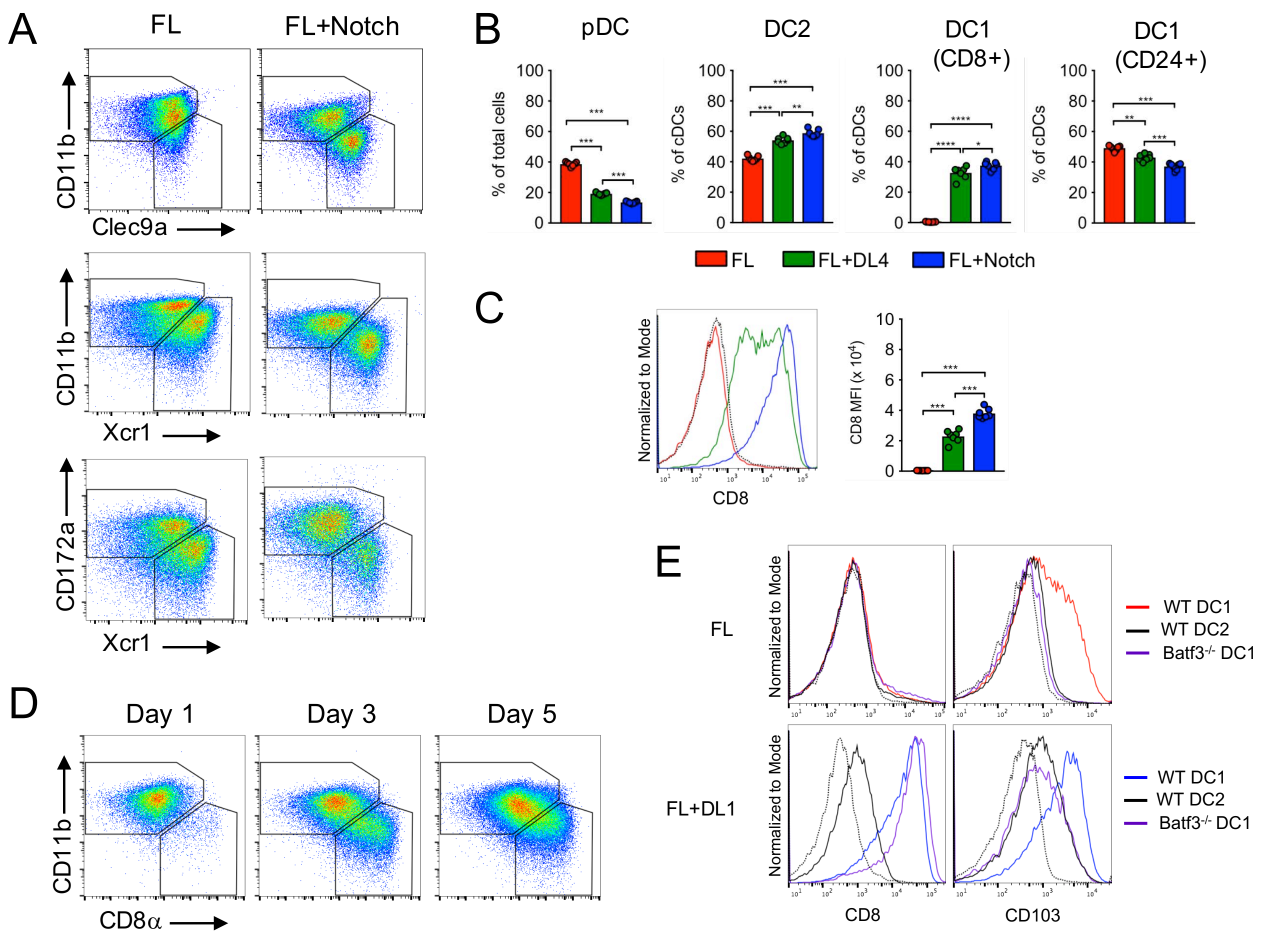

Figure S2. Additional characterization of bone marrow cell differentiation in FL-Notch cultures. Related to Figure 2.

Total murine BM cells were cultured in the presence of Flt3L alone (FL) or Flt3L with OP9 cells expressing the Notch ligand DL1 (FL+Notch). OP9 cells were added on day 3 unless indicated otherwise, and BM cells were analyzed on day 7 of differentiation.

A. Staining of cDC subsets with additional markers. Shown are representative plots of gated B220$^-$ CD11c$^+$ MHC II$^+$ cDC stained for cDC2 markers CD11b or CD172a and cDC1 markers Xcr1 or Clec9a.

B-C. The effect of Notch ligands on DC differentiation. BM cells were differentiated in FL cultures or co-cultures with OP9 expressing DL1 (FL+Notch) or DL4 (FL+DL4). Panel B shows the fraction of DC subsets on day 7. Panel C shows a representative expression profile and averaged mean fluorescence intensity (MFI) of CD8α on gated CD24$^+$ cDC1. Data points represent values in parallel BM cultures from individual mice; bars represent mean.

D. The timing of Notch signaling induction. OP9-DL1 cells were added at days 1, 3 or 5 of FL culture; shown are staining plots of gated B220$^-$ CD11c$^+$ MHC II$^+$ cDC with CD11b$^+$ CD8α$^-$ cDC2 and CD11b$^-$ CD8α$^+$ cDC1 highlighted.

E. The effect of Batf3 deletion on the surface phenotype of cDC1. Shown are representative expression profiles of CD8α and CD103 on gated CD24$^+$ cDC1 from control wild-type (WT) or Batf3-deficient mice. The expression on cDC2 is included as a control; dotted line represents negative staining control.

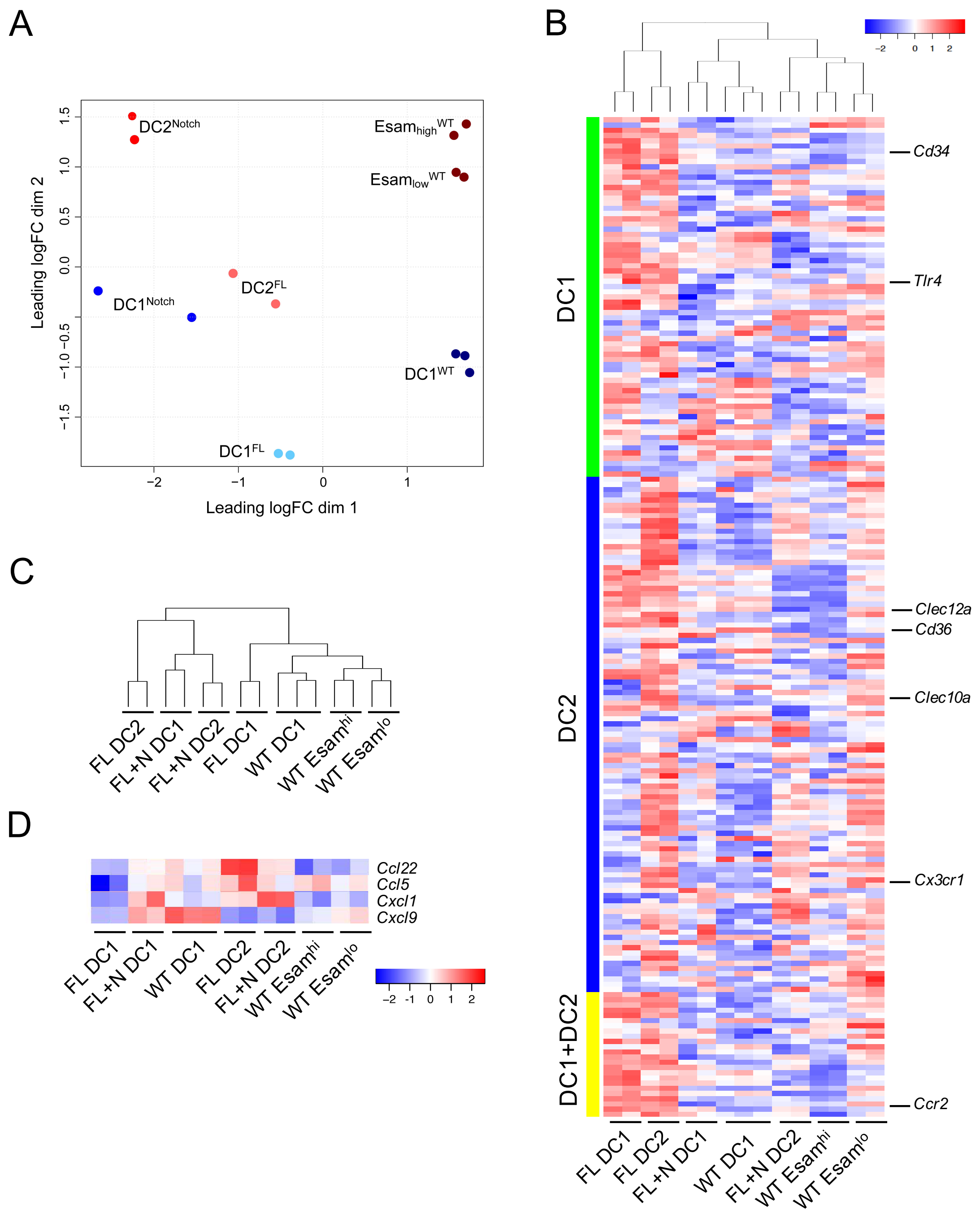

Figure S3. The expression profile of bone marrow-derived DC. Related to Figures 3 and 4.
Duplicate samples of sorted DC subsets from FL and FL-Notch cultures of primary bone marrow were analyzed by RNA-Seq.

A. Multidimensionality scaling (MDS) analysis of RNA-Seq profiles of culture-derived DC and primary splenic DC subsets from wild-type mice (WT). All samples are plotted on the 1st and 2nd dimension of MDS.

B. Heat map of Notch-repressed gene expression in cultured and primary DC subsets. Samples (labeled as in panel A) were hierarchically clustered by the expression of genes that were upregulated preferentially in Notch2-deficient cDC1, cDC2 or both cDC1+cDC2. Select genes are highlighted; color scale represents row Z-score.

C. Unsupervised clustering of cultured and primary splenic DC by the expression of chemokines (Table S4). Shown is the clustering dendrogram with individual replicates of the indicated samples.

D. Heat map of select chemokine expression in cultured and primary splenic DC as determined by RNA-Seq. Color scale represents row Z-score.

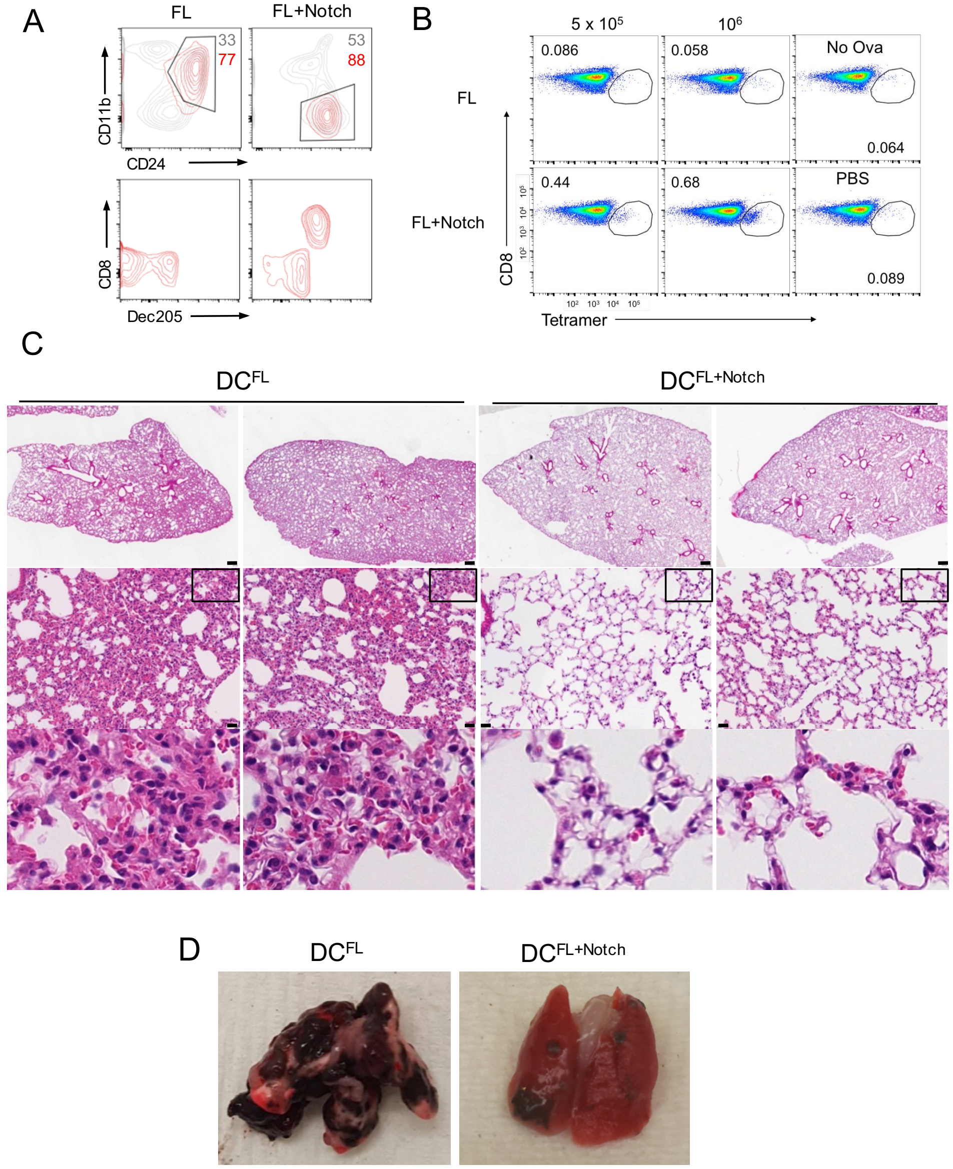

Figure S4. DC-mediated cross-priming and antitumor vaccination. Related to Figure 5.
DC from FL or FL+Notch cultures of primary murine BM were were incubated with OVA, and total DC or enriched cDC1 were injected i.v. into naive wild-type syngeneic recipient mice.
A. Representative cell populations used for in vivo transfer. Top panel shows the overlay of total DC (grey) and magnetically enriched cDC1 (red), with the corresponding $CD24^+$ cDC1 gate highlighted. Bottom panel shows the expression of CD8α and Dec205 in the enriched cDC1.
B. OVA-specific T cells in the spleens of mice that received the indicated number of OVA-pulsed total DC from FL or FL-Notch cultures. Shown are staining profiles of gated $CD44^+$ $TCRb^+$ activated T cells with the $CD8^+$ $tetramer^+$ cells highlighted. Control mice that received FL DC that were not pulsed with OVA (No OVA) or a mock injection (PBS) are also shown.
C. Lung histology of mice that received OVA-pulsed total DC and were subsequently challenged with OVA-expressing B16 melanoma cell line (B16-OVA). Lungs from two representative moribund DC^{FL} recipients (day 14 after B16-OVA injection) or healthy $DC^{FL\text{-}Notch}$ recipients (day 25 after B16-OVA injection) were fixed and stained with H&E. Bars: top row, 200 μm; middle row, 20 μm. Bottom row shows the areas highlighted in the middle row.
D. Gross morphology of the lungs from mice that received OVA-pulsed total DC and were subsequently challenged with B16-OVA. Unlike experiments in Fig. 5D and S4C, B16-OVA was delivered i.v. into the tail vein rather than into the retroorbital sinus. Shown are representative pictures of lungs excised from a moribund DC^{FL} recipient or a healthy $DC^{FL\text{-}Notch}$ recipient on day 28 after B16-OVA injection.

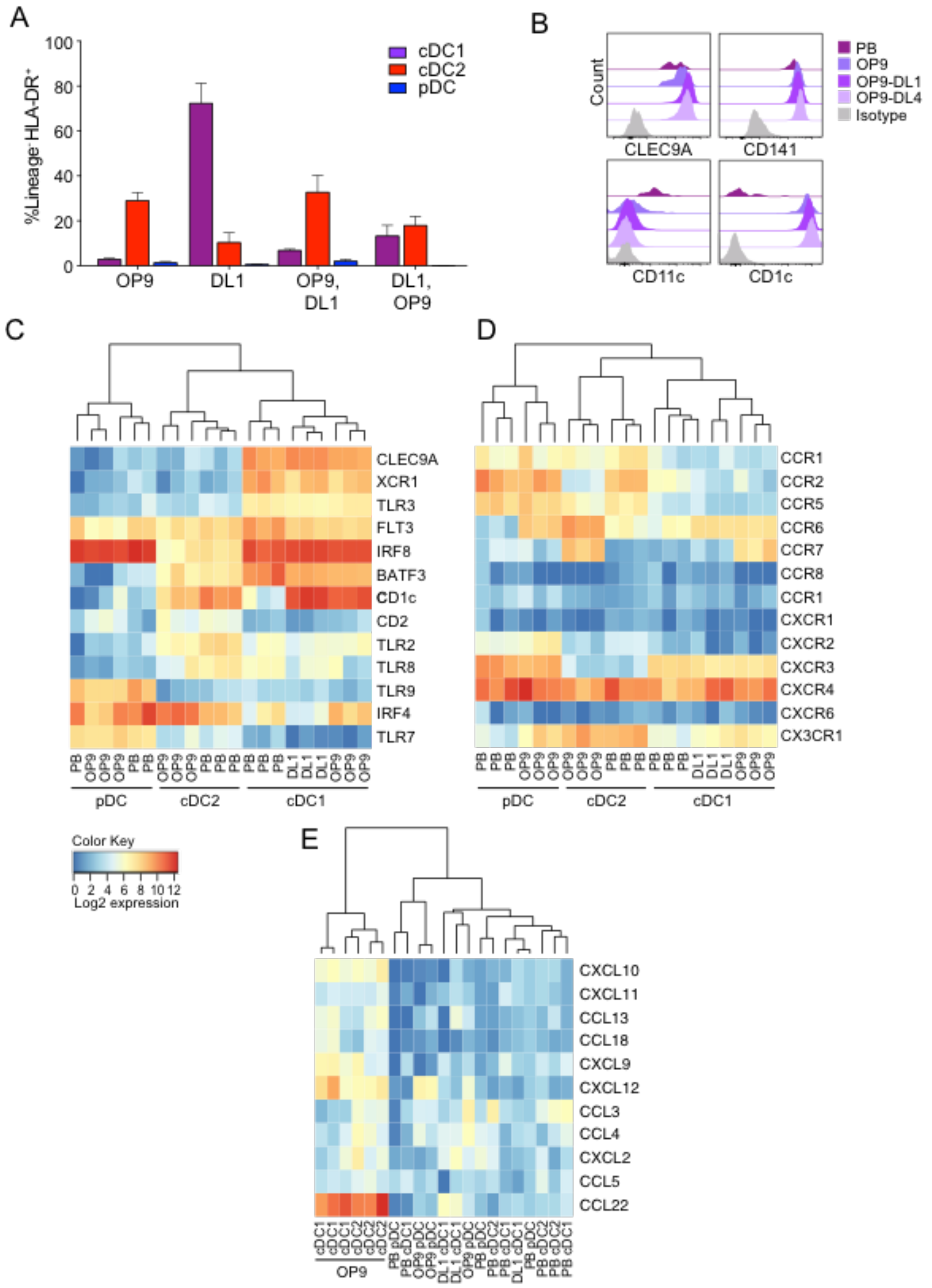

Figure S5, related to Figure 6. Development of cDC1 from human hematopoietic progenitors

Sorted CD34^{+} stem/progenitor cells purified from the human BM were cultured for 2 weeks in the presence of a Flt3L-containing cytokine mix (FSGM) or on monolayers of control OP9, OP9-DL1 or OP9-DL4 cells.

A. The effect of the timing of OP9-DL1 addition. Cells were cultured on OP9 (n=9) or OP9-DL1 (DL1; n=7) for the entire culture period, or replated from OP9 to OP9-DL1 (OP9,DL1;n=3) or from OP9-DL1 to OP9 (DL1,OP9; n=2) after 1 week. Shown is the proportion of indicated DC subsets out of total Lin^{-} HLA-DR^{+} cells (mean ± S.E.M.)

B. The expression of DC markers. Shown are representative staining profiles of cDC1 from blood (PB) or culture with OP9, OP9-DL1 or OP9-DL4. Antigen expression is shown relative to isotype control (grey).

C-E. Heatmaps of DC subset specific surface antigen, TLR and transcription factor (**C**), chemokine receptor (**D**) or chemokine (**E**) gene expression in primary DC or DC cultured on OP9 or OP9-DL1 (DL1), as determined by NanoString nCounter analysis (Human Immunology_V2 panel plus 30 genes). Hierarchical clustering of samples by expression of indicated genes is shown. Color scales represent log2 expression.
